# EWSR1-induced circNEIL3 promotes glioma progression and exosome-mediated macrophage immunosuppressive polarization via stabilizing IGF2BP3

**DOI:** 10.1186/s12943-021-01485-6

**Published:** 2022-01-14

**Authors:** Ziwen Pan, Rongrong Zhao, Boyan Li, Yanhua Qi, Wei Qiu, Qindong Guo, Shouji Zhang, Shulin Zhao, Hao Xu, Ming Li, Zijie Gao, Yang Fan, Jianye Xu, Huizhi Wang, Shaobo Wang, Jiawei Qiu, Qingtong Wang, Xing Guo, Lin Deng, Ping Zhang, Hao Xue, Gang Li

**Affiliations:** 1 Department of Neurosurgery, Qilu Hospital, Cheeloo College of Medicine and Institute of Brain and Brain-Inspired Science, Shandong University, 107 Wenhua Western Road; Jinan, Shandong 250012, China, Jinan, 250012 Shandong China; 2 Shandong Key Laboratory of Brain Function Remodeling, Jinan, Shandong China; 3 Department of Neurosurgery, The Affiliated Yantai Yuhuangding Hospital of Qingdao University, Yantai, Shandong China; 4 Department of Neurosurgery, Taian Central Hospital, Taian, Shandong China

**Keywords:** Glioma, circNEIL3, IGF2BP3, Tumour microenvironment, Tumour-associated macrophages, Exosome

## Abstract

**Background:**

Gliomas are the most common malignant primary brain tumours with a highly immunosuppressive tumour microenvironment (TME) and poor prognosis. Circular RNAs (circRNA), a newly found type of endogenous noncoding RNA, characterized by high stability, abundance, conservation, have been shown to play an important role in the pathophysiological processes and TME remodelling of various tumours.

**Methods:**

CircRNA sequencing analysis was performed to explore circRNA expression profiles in normal and glioma tissues. The biological function of a novel circRNA, namely, circNEIL3, in glioma development was confirmed both in vitro and in vivo. Mechanistically, RNA pull-down, mass spectrum, RNA immunoprecipitation (RIP), luciferase reporter, and co-immunoprecipitation assays were conducted.

**Results:**

We identified circNEIL3, which could be cyclized by EWS RNA-binding protein 1(EWSR1), to be upregulated in glioma tissues and to correlate positively with glioma malignant progression. Functionally, we confirmed that circNEIL3 promotes tumorigenesis and carcinogenic progression of glioma in vitro and in vivo. Mechanistically, circNEIL3 stabilizes IGF2BP3 (insulin-like growth factor 2 mRNA binding protein 3) protein, a known oncogenic protein, by preventing HECTD4-mediated ubiquitination. Moreover, circNEIL3 overexpression glioma cells drives macrophage infiltration into the tumour microenvironment (TME). Finally, circNEIL3 is packaged into exosomes by hnRNPA2B1 and transmitted to infiltrated tumour associated macrophages (TAMs), enabling them to acquire immunosuppressive properties by stabilizing IGF2BP3 and in turn promoting glioma progression.

**Conclusions:**

This work reveals that circNEIL3 plays a nonnegligible multifaceted role in promoting gliomagenesis, malignant progression and macrophage tumour-promoting phenotypes polarization, highlighting that circNEIL3 is a potential prognostic biomarker and therapeutic target in glioma.

**Supplementary Information:**

The online version contains supplementary material available at 10.1186/s12943-021-01485-6.

## Background

Glioma is the most common and aggressive primary malignancy of the central nervous system and has a poor prognosis. Glioblastoma multiforme (GBM) is a kind of brain glioma, with a WHO grade of IV and the highest degree of malignancy [1, 2]. Even if active surgery is combined with radiotherapy and chemotherapy, the treatment effect of glioma is still poor [3, 4]. Thus, it is important to clarify the molecular mechanism of glioma progression to improve the prognosis of patients.

Circular RNAs (circRNAs), a special class of noncoding RNAs, do not have a 5′ end cap or a 3′ end poly(A) tail; rather, they form a circular structure by covalent bonds and are characterized by high stability, abundance, and conservation [5, 6]. Mounting evidence has shown that circRNAs play key roles in various types of cancers [7, 8], including glioma [9, 10]. Although there are a large number of circRNAs that appear capable of regulating gliomagenesis, the function of a handful of others remains to be determined.

The impact of abnormal glioma-associated signalling is not limited to cancer cells but rather extends to the complex network of interactions with the tumour microenvironment (TME). The function and composition of the glioma TME are influenced by the intrinsic signalling pathways and secretory factors of cancer cells. In turn, cells in the TME can maintain the cancer hallmark by regulating a variety of tumour biological characteristics, including cell proliferation, survival, migration and immune escape [11, 12]. Within the glioma microenvironment, tumour-associated macrophages (TAMs), composed of bone marrow-derived macrophages (BMDMs) and brain-resident microglia (MG), constitute the most abundant cell population in the glioma TME [13, 14] and have been revealed as critical cell components for gliomagenesis and malignant progression [12, 15]. Several studies have shown that exosomes (50–1150 nm), derived from multivesicular bodies (MVBs) that contain proteins, lipids, and nucleic acids, such as miRNAs, lncRNAs, and circRNAs, can act as signalling vehicles with multiple functions in the interaction between glioma cells and TME cells [16, 17]. Mounting evidence has shown that circRNAs are involved in the multifaceted regulation of the TME [18, 19]. However, details on the mechanisms by which circRNAs in exosomes regulate TAMs in glioma have remained elusive until now. Understanding these multifaceted communications between gliomas and TAMs could open new avenues for therapy.

In the present study, we identified a circRNA derived from NEIL3, hsa_circ_0001460, named circNEIL3, by analysing the expression profiles of circRNAs in glioma tissues. The expression of circNEIL3, which can be regulated by EWS RNA-binding protein 1 (EWSR1), increased with the increasing grade of gliomas. Functionally, we confirmed that circNEIL3 promoted tumorigenesis and the progression of glioma in vitro and in vivo. Mechanistically, circNEIL3 stabilizes IGF2BP3 protein, a known oncogenic protein, by preventing HECTD4-mediated ubiquitination. Moreover, circNEIL3-overexpressing glioma cells drive macrophage infiltration into the TME by activating YAP1 signalling and secreting CCL2 and LOX, which are strong macrophage chemokines. Finally, circNEIL3 could be packaged into exosomes by hnRNPA2B1 and transmitted to infiltrating TAMs, thereby enabling them to acquire immunosuppressive properties by stabilizing IGF2BP3, in turn promoting glioma progression. Our data thus provide evidence that circNEIL3 may serve as a potential prognostic biomarker and therapeutic target in glioma.

## Materials and methods

### Patient specimens and public patient cohorts

Thirty-nine human glioma tissues and 8 NBTs for circRNA sequencing and RNA sequencing were obtained from patients admitted to Qilu Hospital from November 2017 to October 2019. All participants provided written informed consent, and the study was approved by the Ethics Committee on Scientific Research of Shandong University Qilu Hospital (approval number: KYLL-2018-324). The detailed clinicopathological characteristics are described in Additional file Table S8.

The RNA-seq transcriptome data and corresponding clinicopathological parameters of the TCGA glioma dataset were obtained from the TCGA database (http://cancergenome.nih.gov/). The CGGA RNA-seq dataset and the corresponding clinicopathological parameters were obtained from the CGGA database (http://www.cgga.org.cn/). The circRNA sequencing and mRNA sequencing data of our local samples have been deposited in the Genome Sequence Archive (GSA) under accession number CRA002339, and data were released when the paper was published. The processed data are available from the corresponding author upon reasonable request.

### Cell lines and culture

Human glioma cell lines (A172 and U251) were purchased from the Culture Collection of the Chinese Academy of Sciences (Shanghai, China) and cultured in Dulbecco’s modified Eagle medium (DMEM; Thermo Fisher Scientific; USA) supplemented with 10% foetal bovine serum (FBS; Gibco; USA). All patient-derived GSC cell lines and neural progenitor cells (NPCs) were kindly donated by Dr. Frederick F Lang and Dr. Krishna P.L. Bhat (The University of Texas, M.D. Anderson Cancer Center, Houston, TX) and cultured in DMEM/F12 (10,565,018; Gibco, USA) supplemented with 2% B-27 without serum supplement (17,504,044; Gibco, USA), 20 ng/mL human recombinant EGF (236-EG; R&D Systems, USA) and 20 ng/mL human recombinant bFGF (233-FB; R&D Systems). All cell lines were maintained in a humidified chamber containing 5% CO2 at 37 °C.

### Quantitative real-time PCR (qRT-PCR)

Total RNA was extracted from glioma cells using TRIzol reagent (Life Technologies, Carlsbad, CA, USA). Reverse transcription was performed using 1 μg of total RNA and the High Capacity cDNA Reverse Transcription Kit (Toyobo, FSQ-101, Shanghai, China) according to the manufacturer’s protocol. An Mx-3000P Quantitative PCR System (Applied Biosystems, Foster City, USA) was used for qRT-PCR. The expression of GAPDH was used as a control to calibrate the original mRNA concentrations in tissues and cells. Target gene expression was calculated using the 2 ^-ΔΔCT^ method. The primer sequences are detailed in the Additional file Table S9.

### RNase R treatment

Total RNA (2 μg) was incubated for 15 min at 37 °C with 5 U/μg RNase R (Epicentre Technologies, Madison, WI, USA) and then analysed by qRT-PCR.

### Actinomycin D assays

U251 and A172 cells were transferred to six-well plates, exposed to 2 μg/ml actinomycin D and collected at the indicated time points. The stability of circNEIL3 and NEIL3 was analysed by qRT-PCR.

### Lentivirus, siRNA, plasmid construction, and cell transfection

Human full-length circNEIL3 and sh-circNEIL3 plasmids were selected and inserted into the pLVX-IRES-Puro vector for stable overexpression and knockdown, respectively, with empty plasmid used as a control. si-circNEIL3, si-IGFB2P3, si-EWSR1 and si-HECTD4 were purchased from Boshang (Jinan, China). FLAG-tagged expression vectors for full-length IGF2BP3 and site-directed mutants (K450R) were provided by Boshang (Jinan, China). For the transfection of siRNAs and plasmids, cells were transfected using the Lipofectamine 3000 kit (Invitrogen, Carlsbad, CA, USA) according to the manufacturer’s instructions. All sequences are listed in Additional file Table S10.

### In vitro cell behaviour assays

#### Wound healing assay

In all, 8X10^5^ cells were seeded in 6-well plates, grown to confluency, and scratched with a 200 μl pipette tip to make wounds of a consistent length before they were cultured in basal medium. After the cells were washed with PBS to remove dissociated cellular fragments, each wound was imaged at 0 and 24 h by inversion microscopy (Olympus, Japan). Cell migration was calculated using ImageJ.

#### Transwell assay

In all, 3X10^4^ cells were seeded in the upper layer of the Transwell membrane, while the lower chamber contained 10% foetal bovine serum to induce cell migration. After the Transwells were incubated at 37 °C under an atmosphere with 5% CO2 for 24 h, the upper layer of the Transwell membrane was wiped, and the cells that passed through the membrane were stained with crystal violet for 20 min and observed by microscopy.

#### CCK-8 assay

We seeded cells in 96-well plates at 2 × 10^3^ per well in 100 μl of complete medium and 10 μl of CCK-8 reagent (RiboBio, Guangzhou, China) for 1 h each day after 3 days of culture. We then used a microplate to measure the absorbance of each well at 450 nm. Each sample was evaluated in triplicate.

#### EdU assay

We used an EdU cell proliferation assay kit (RiboBio, Guangzhou; China). Cells were incubated with 200 μl of 5-ethynyl-20-deoxyuridine for 2 h at 37 °C and then fixed in 4% paraformaldehyde for 20 min. After that, the cells were permeabilized with 0.4% Triton X-100 for 10 min and incubated with Apollo® reagent (100 μl) for 30 min. Finally, the cells were stained with Hoechst for 30 min, and representative images were obtained using a Nikon inverted fluorescence microscope. The cell proliferation rate was calculated using the ratio of EdU-positive cells (red) to Hoechst-positive cells (blue).

#### Colony formation assay

In all, 2000 cells were seeded in 6-well plates and cultured in complete medium supplemented with 10% FBS. After 14 days, colonies were stained using crystal violet (Solarbio, Beijing, China) for 30 min and washed with PBS three times. Finally, we counted colonies with diameters greater than 1 mm.

#### Neurosphere formation assay

GSCs were dispersed in Accutase solution and seeded in 6-well plates at 2000 cells per well. Microscopy was used to view images and measure the sphere diameters for quantification analysis.

#### Extreme limiting dilution assay (ELDA)

GSCs were seeded in 96-well plates at densities of 0, 2, 4, 8, 16, 32 and 64 cells per well with 10 replicates per condition. After 14 days, the number of wells in which cells grew into colonies was calculated.

### In vivo tumorigenesis and metastasis assays

To investigate the roles of circNEIL3 in vivo, tumour xenografts were established in 4-week-old male BALB/c nude mice purchased from the Model Animal Research Centre of Nanjing University (Nanjing, China). U251 luciferase cells (2X10^5^) with overexpression or knockdown of circNEIL3 and their respective empty vectors were implanted into the brains of nude mice. Bioluminescence imaging was used to image the mouse brains every 4 days after cell implantation. Next, we randomly chose 10 mice in each group and sacrificed them on the same day (21 days). The brains were fixed with paraformaldehyde for further study. The remaining mice (10/group) were kept until death for survival analysis. All procedures that involved mice were approved by the Animal Care and Use Committee of the Qilu Hospital of Shandong University.

### Western blot assays

Protein was extracted from U251 cells. Protein lysates were loaded and separated by SDS-PAGE before they were transferred to polyvinylidene difluoride (PVDF) membranes. The blots were incubated with primary antibodies against IGF2BP3, CDK6, CDK4, FLAG, c-myc, spp1, EWSR1, HECTD4, and hnRNPA2B1. Antibody information is listed in Additional file Table S11.

### Flow cytometry

Anti-CD163-PE and anti-CD11b-APC antibodies were used to stain cells to detect CD11b + CD163+ macrophages. Isotype controls were run in parallel. Flow cytometry was performed by using a BD Accuri C6 flow cytometer (BD Biosciences). Antibody information is listed in Additional file Table S11.

### RNA FISH-immunofluorescence microscopy

U251 and A172 cells were fixed in 4% paraformaldehyde for 15 min, washed with PBS and then treated with pepsin (1% in 10 mM HCl). For denaturation, cells were dried and incubated with 20 nM FISH probe in hybridization buffer at 73 °C for 5 min. Hybridization was performed at 37 °C overnight. Finally, the slides were washed, dehydrated, and stained with DAPI prior to imaging. The RNA FISH probe was designed and synthesized by GenePharma (Shanghai, China). The sequences are listed in Additional file Table S11.

### Co-immunoprecipitation (co-IP)

U251 and A172 cells were lysed in 500 μl of co-IP buffer (Millipore, Billerica, MD, USA) with PMSF. The lysates were centrifuged at 12000×g for 15 min. The supernatant was mixed with magnetic beads and incubated with antibodies at 4 °C for 12 h. After that, the beads were washed with co-IP buffer 4 times. Finally, the proteins were eluted for mass spectrometry and Western blot analysis.

### RNA immunoprecipitation (RIP)

A Magna RIP RNA-binding Protein Immunoprecipitation Kit (Millipore, Billerica, MA, USA) was used to perform RIP experiments according to the manufacturer’s instructions. U251 and A172 cells (1 × 10^7^) were lysed in 1 ml of RIP lysis buffer with RNase inhibitors. The cell lysates were then incubated with beads coated with IgG, anti-IGFB2P3 or ant-hnRNAPA2B1 antibodies (Millipore) on a rotator at 4 °C overnight. RNA was extracted with an RNeasy MinElute Cleanup Kit (Qiagen, Valencia, CA, USA) and then detected by qRT-PCR.

### RNA pull-down assays

Biotinylated circNEIL3 and its anti-sense sequence were synthesized by RiboBio (GenePharma, Shanghai, China). U251 and A172 cells were lysed and incubated with a biotin-labelled circNEIL3 probe. Then, cell lysates were incubated with streptavidin-conjugated agarose magnetic beads (Thermo Fisher Scientific; Waltham, MA, USA) at room temperature. Finally, interacting proteins were identified by Western blot and mass spectrometry.

### Immunohistochemistry (IHC)

Tissues were incubated with 4% paraformaldehyde, embedded in paraffin, and then sliced into slices. We used primary antibodies against CD44 and Ki67. Antibodies information were listed in listed in Additional file Table S11. After primary and secondary antibody incubations, HRP-labelled streptavidin solution was added to the samples for 15 min. The immunocomplex was visualized with DAB, and the nuclei were counterstained with haematoxylin. Then, we used a microscope (Leica, Wetzlar, Germany) to take pictures.

### Immunofluorescence (IF)

U251 and A172 cells (2000 per well) were seeded in μ-Slide 8 wells overnight. Cells were then fixed with 4% paraformaldehyde for 20 min and treated with 0.1% Triton X-100 in PBS for 10 min. Cells were blocked with 5% BSA for 30 min at 37 °C, incubated with primary antibody overnight at 4 °C, and washed with PBS three times. Then, the cells were incubated with DAPI for 30 min. All images were viewed under a LeicaSP8 confocal microscope (Leica Microsystems, Wetzlar, Germany).

### TME cell infiltration characteristics

The stromal score, immune score, ESTIMATE score, and tumour purity were analysed by the ESTIMATE algorithm based on the RNA-seq data. We then used the ssGSEA algorithm to quantify the enrichment score of each sample immune cell infiltration in the glioma TME. The gene sets (including immune cell types, immune-related pathways and functions) were obtained from Bindea et al. [20]. And the TAM BMDM and TAM MG gene sets were obtained from Bowman et al. [21].

### Differential expression analysis and functional analysis

We classified patients into two groups based on the median cut-off expression of circNEIL3 in our local dataset or IGF2BP3 in the TCGA glioma dataset. Then, differential expression analysis was analysed using the R package “DEseq2”. Furthermore, upregulated genes were analysed using the web tool “Metascape” for pathway and process enrichment analysis (https://metascape.org/gp/index.html).

To explore the biological behaviours among these distinct circNEIL3 and IGF2BP3 expression samples, we used some gene sets of HALLMARK [22] from the MSigDB database to estimate pathway enrichment scores for each sample for GSVA enrichment analysis using the “GSVA” R package. GSEA was also used to estimate the enrichment of various biological processes in each sample.

### Statistical analysis

Statistical analyses were performed using GraphPad Software 8 (GraphPad Software Inc., CA, USA). The survival curves for the prognostic analysis were generated via the Kaplan–Meier method, and log-rank tests were utilized to identify the significance of the differences. The specificity and sensitivity of circNEIL3 were assessed via receiver operating characteristic (ROC) curves, and the area under the curve (AUC) was quantified using the pROC R package. The correlation was analysed by pearson correlation test. All data are expressed as the means ± standard deviation (SD). Student’s t-test was used for two-group comparisons. For comparisons among more than two groups, the Wilcoxon test and one-way ANOVA were used for non-parametric and parametric data. *P* > 0.05 was considered not significant (ns), and *P* < 0.05 was considered statistically significant (*P < 0.05; **P < 0.01; ***P < 0.001, ****P < 0.0001). All data processing with R packages was performed using R Studio Software (version 3.6.3).

## Results

### CircNEIL3 was significantly upregulated in glioma tissues

**Fig. 1:**
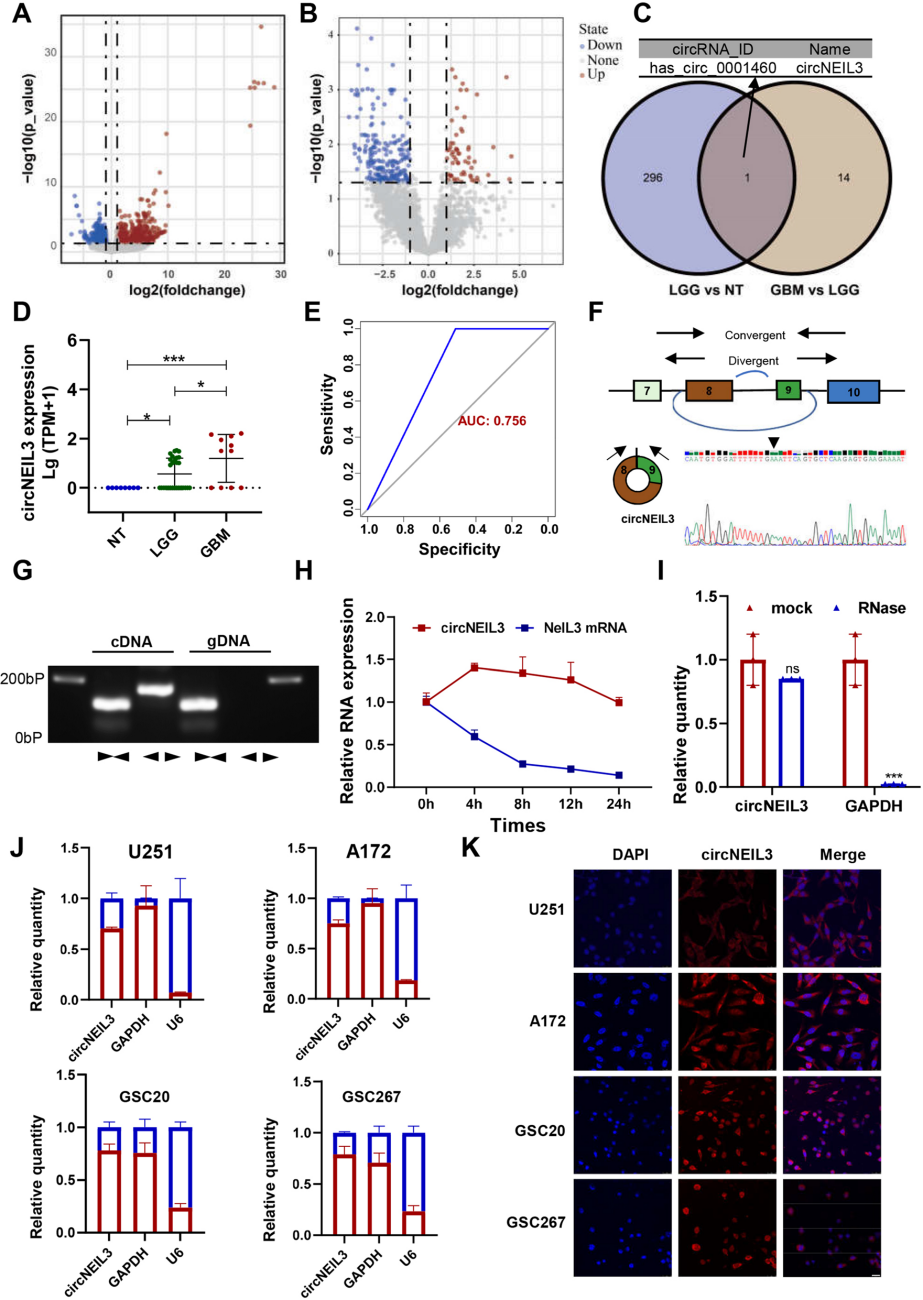
CircRNA expression profiles in glioma and characterization of circNEIL3. **A** Volcano plots of circRNAs that were differentially expressed between LGG tissues and NBTs. **B** Volcano plots of circRNAs that were differentially expressed between GBM samples and LGG samples. **C** Venn diagram showing the overlapping upregulated circRNAs (Log2 FC > 4) between LGG tissues and NBTs and between GBM and LGG tissues. **D** Relative expression of circNEIL3 in NBTs, LGG tissues and GBM tissues detected by high-throughput sequencing. **E** An ROC curve was used to assess the diagnostic value of circNEIL3 for glioma. **F** Schematic illustration of the genomic location and back splicing of circNEIL3 with the splicing site validated by Sanger sequencing. **G** RT-PCR with divergent and convergent primers and agarose gel electrophoresis analysis were performed to detect the presence of circNEIL3 and its maternal gene NEIL3 in cDNA and gDNA samples from GBM cells. **H** Actinomycin D treatment was used to evaluate the stability of circNEIL3 and NEIL3 mRNA in GBM cells. **I** RNase treatment was used to evaluate the stability of circNEIL3 and NEIL3 mRNA in GBM cells. **J** Nuclear-cytoplasmic fractionation assays showed that circNEIL3 was mostly localized in the cytoplasm of GBM cells. **K** RNA FISH assays showing the cellular localization of circNEIL3 in GBM cells. The circNEIL3 probe was labelled with Cy3 (red), while nuclei were stained with DAPI (blue). Scale bar, 25 μm. All data are presented as the means ± SD, ns, *P* > 0.05, *P < 0.05, **P < 0.01, ***P < 0.001, ****P < 0.0001

To identify potential oncogenic circRNAs involved in the tumorigenesis and progression of glioma, we analysed the expression profiles of circRNAs in 39 glioma tissues (11 GBM cases and 28 low-grade glioma (LGG) cases) and 8 normal brain tissues (NBTs) using RNA sequencing (RNA-seq). Volcano plots showed systemic differences in circRNA expression between GBM and LGG, as well as between LGG and NBTs (Fig. 1A, B, Log2Fold Change (FC) ≥ 1, Padj≤0.05). We further analysed the differentially upregulated circRNAs in glioma tissues (Log2FC ≥ 4, Padj≤0.05) and identified hsa_circ_0001460, the only circRNA that was upregulated with increasing glioma grade (Fig. 1C). We named hsa_circ_0001460 as circNEIL3, which is generated from NEIL3. CircNEIL3 was markedly upregulated in glioma tissues compared with NBTs, and its expression increased with the increasing glioma grade (Fig. 1D). ROC (Receiver operating characteristic) curve was used to assess the diagnostic efficacy of circNEIL3 in glioma grade, the AUC (area under the curve) of which was 0.719, suggesting that circNEIL3 could predict poor prognosis in glioma patients (Fig. 1E).

Through the UCSC Genomics Institute Bioinformatics site (http://genome.ucsc.edu/), we identified that circNEIL3 was backspliced between exon 8 and exon 9 of the NEIL3 gene with a length of 596 nt (Fig. 1)F. The back-splice junction site of circNEIL3 was amplified using divergent primers and was confirmed by Sanger sequencing (Fig. 1F). Furthermore, we designed divergent and convergent primers to amplify circNEIL3 and its linear form. The results of agarose gel electrophoresis analysis of the RT-qPCR products showed that circNEIL3 could only be amplified from cDNA, while its linear form could be amplified from cDNA and gDNA (Fig. 1G). Moreover, we confirmed that circNEIL3 was more stable than NEIL3 by treatment with RNase R and actinomycin D (Fig. 1H, I). The functions of circRNAs are mostly related to their intracellular localization. We then performed nuclear-cytoplasmic fractionation experiments and FISH assays. The results showed that circNEIL3 was mostly localized to the cytoplasm (Fig. 1J, K). In summary, these results indicated that circNEIL3 was significantly upregulated in glioma tissues, and its expression was highest in GBM, suggesting that it is involved in tumorigenesis and the malignant progression of glioma.

### CircNEIL3 promoted glioma tumorigenesis in vitro and vivo

To investigate the potential biological role of circNEIL3 in the progression of glioma, we performed KEGG enrichment analysis on genes that were significantly positively correlated with circNEIL3 expression (Additional file Table S1) based on our transcriptome sequencing data. As shown in Fig. S1A, the genes were significantly enriched in many pathways involved in tumour pathogenesis, including cell proliferation regulation pathways (such as cell cycle and DNA replication), pathways associated with tumour invasion and migration (such as focal adhesion and ECM-receptor interaction), and classical signalling pathways involved in tumorigenesis, including p53 signalling pathways and pathways in cancer. Gene set enrichment analysis (GSEA) also showed that these pathways were significantly enriched in the high circNEIL3 expression group (Fig. S1B). Furthermore, to explore the biological behaviours among these distinct circNEIL3 expression samples, we used a single-sample GSEA (ssGSEA) algorithm to estimate pathway enrichment scores for each sample (see Methods). As shown in Fig. S1C, compared to the low circNEIL3 expression group, the high expression group was more markedly enriched in carcinogenic signalling pathways.

**Fig. 2:**
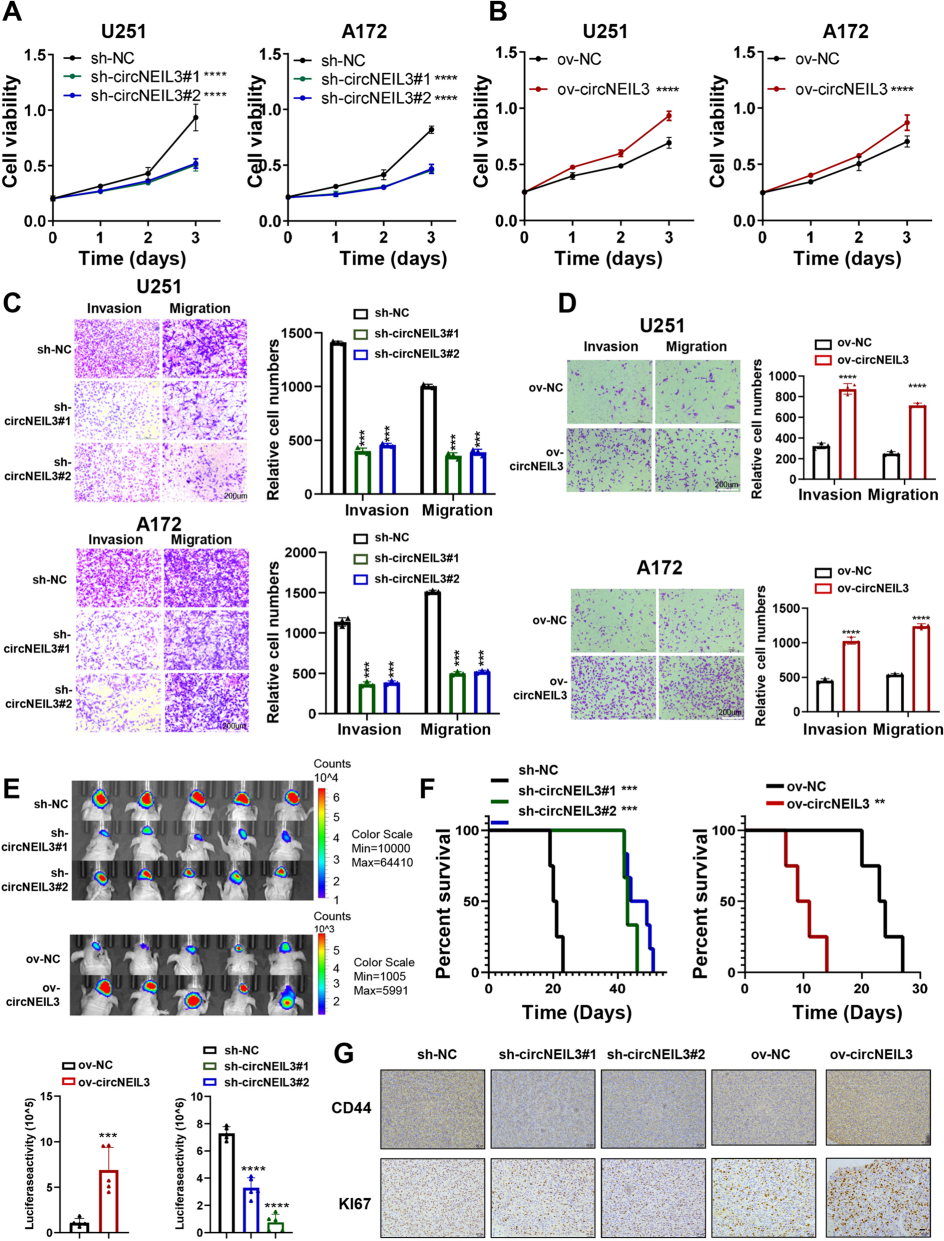
CircNEIL3 promotes proliferation and metastasis of GBM cells in vitro and vivo. CCK8 assays showing the proliferation ability of GBM cells transfected with **(A)** sh-NC or sh-circNEIL3, and **(B)** ov-NC or ov-NEIL3, *n* = 3. Representative transwell migration and invasion assays showing the migration and invasion ability of GBM cells transfected with **(C)** sh-NC or sh-circNEIL3, and **(D)** ov-NC or ov-NEIL3. Quantification histogram represented relative cell numbers, n = 3, scale bar, 200um. **E** Up, bioluminescent image showing the tumor size for animals in different groups in the indicated time. *n* = 5 for each group. Down, statistical analysis of bioluminescent tracking plots. **F** Kaplan-Meier survival curves for animals in different groups, n = 5 for each group. **G** Represented CD44 and KI67 immunohistochemistry images for a subgroup of animal sacrificed simultaneously in each group, n = 5 for each group, scale bar, 20um. All data are presented as the means ± SD, **p < 0.01, ***p < 0.001, ****p < 0.0001

Since circNEIL3 was potentially involved in cell proliferation, invasion and migration, we further investigated the function of circNEIL3 in cell behaviour. Consistent with our bioinformatics analysis results, we confirmed that downregulation of circNEIL3 significantly inhibited cell proliferation, migration, and invasion. Overexpression of circNEIL3 remarkably promoted these cellular behaviours in vitro (Fig. 2A-D, Fig. S2A-F). We also demonstrated that circNEIL3 significantly promoted the tumorsphere expansion (Fig. S2G-H) and sphere formation ability of the GSCs (Figs. S2I-J). Moreover, in vivo experiments revealed that circNEIL3 downregulation could markedly inhibit the tumour growth and invasiveness and prolong the survival of tumour-bearing mice, while circNEIL3 overexpression elicited the opposite effects (Fig. 2E, F, and Fig. S2K). In addition, immunohistochemistry (IHC) of the excised tumour sections indicated that the expression of Ki67 (a proliferation marker) and CD44 (an invasiveness marker) in circNEIL3-knockdown tumour tissues was lower than that in the vector group, while circNEIL3 overexpression showed the opposite effects (Fig. 2G). Taken together, our data suggest that circNEIL3 is functionally important in regulating tumorigenesis and glioma progression.

### EWSR1 promotes the biogenesis of circNEIL3 in glioma

**Fig. 3:**
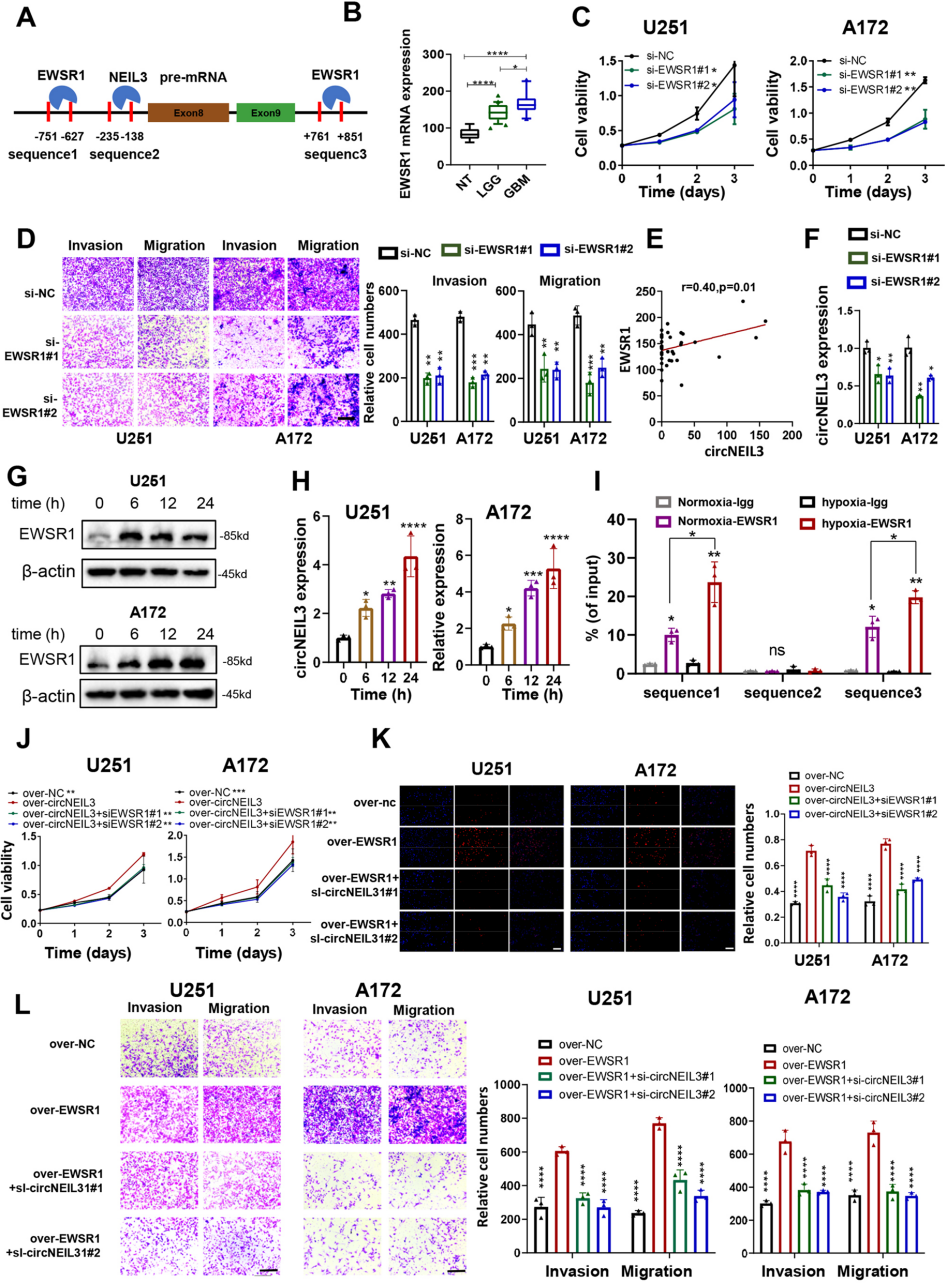
EWSR1 promotes the biogenesis of circNEIL3. **A** The binding sites of EWSR1 were predicted in the upstream and downstream region of NEIL3 pre-mRNA transcript using the starbase and circinteractome database. **B** Relative expression of EWSR1 in our local NBTs, LGG and GBM tissues detected by high-throughput sequencing. **C** CCK8 assays showing the proliferation ability of GBM cells transfected with sh-NC or sh-circNEIL3, *n* = 3. **D** Representative transwell migration and invasion assays showing the migration and invasion ability of GBM cells transfected with sh-NC or sh-circNEIL3. Quantification histogram represented relative cell numbers, n = 3, scale bar, 200um. **E** The correlation between circNEIL3 and EWSR1 expression in our local glioma high-throughput sequencing data (*n* = 39). **F** qRT-PCR assays showed the expression of circNEIL3 in GBM cells transfected with sh NC or sh EWSR1, n = 3. **G** Western blot assays showing the expression of EWSR1 under hypoxia treatment for 0, 6, 12, 24 h. **H** qRT-PCR assays showing the expression of circNEIL3 under hypoxia treatment for 0, 6, 12, 24 h, *n* = 3. **I** RIP and qRT-PCR assays were performed to verify the putative binding site of NEIL3 pre-mRNA with EWSR1 under normal and hypoxia environment, n = 3. **(J)** CCK8 assays, **(K)** Representative EDU assays showing the proliferation ability of GBM cells co-transfected with ov-NC, ov-EWSR1 and si-circNEIL3 as indicated. Quantification histogram represented relative cell numbers, n = 3, scale bar = 50um. **L** Representative transwell migration and invasion assays showing the migration and invasion ability of GBM cells co-transfected with ov-NC, ov-EWSR1 and si-circNEIL3 as indicated, Quantification histogram represented relative cell numbers, n = 3, scale bar, 200um. All data are presented as the means ± SD, and the ov-EWSR1 group is indicated as the control in (J-L), ns, *P* > 0.05, *P < 0.05, **P < 0.01, ***P < 0.001, ****P < 0.0001

The mechanism of cyclization of circRNAs is regulated by RNA-binding proteins (RBPs) binding to the upstream and downstream regions of pre-mRNA [23, 24]. We predicted three binding sites of EWSR1 in the upstream and downstream regions of circNEIL3 called sequence 1, sequence 2 and sequence 3 (Fig. 3A) by using the CircInteractome database (https://circinteractome.nia.nih.gov/) and starBase 3.0 (http://starbase.sysu.edu.cn/index.php) database (Fig. S3A, B). We found that the EWSR1 expression level increased with the grade of glioma, and patients with high expression of EWSR1 had a worse prognosis than those with low expression (Fig. 3B, Fig. S3D, E). Furthermore, we validated that knocking down EWSR1 could inhibit GBM cell proliferation, invasion and migration (Fig. 3C, D, and Fig. S3F). In our own database, we found that circNEIL3 is positively correlated with EWSR1 (Fig. 3E), and the expression of circNEIL3 is significantly deceased in U251 and A172 cells with EWSR knockdown (Fig. 3F). A recent study indicated that EWSR1 could be more active in hypoxic environments [25]. Thus, we subjected U251 and A172 cells to hypoxic conditions for 0 h, 6 h, 12 h and 24 h, and the expression of EWSR1 and circNEIL3 were upregulated as the exposure time increased (Fig. 3G, H). RIP-qPCR assays confirmed that EWSR1 could bind to sequence 1 and sequence 3 of NEIL3 pre-mRNA, and the binding ability was significantly upregulated under hypoxic conditions (Fig. 3I). To further confirm that the observed EWSR1-mediated phenotypes were facilitated by the dysregulation of circNEIL3 expression, we performed functional rescue assays. As shown in Fig. 3J-L, EWSR1 overexpression induced increases in proliferation, migration and invasion in U251 and A172 cells, which could be reversed by circNEIL3 downregulation. Our data demonstrated that circNEIL3 could be cyclized by EWSR1 and that this process was more active under hypoxic conditions.

### CircNEIL3 physically interacts with IGF2BP3 and inhibits the ubiquitin/proteasome-mediated degradation of IGF2BP3

**Fig. 4:**
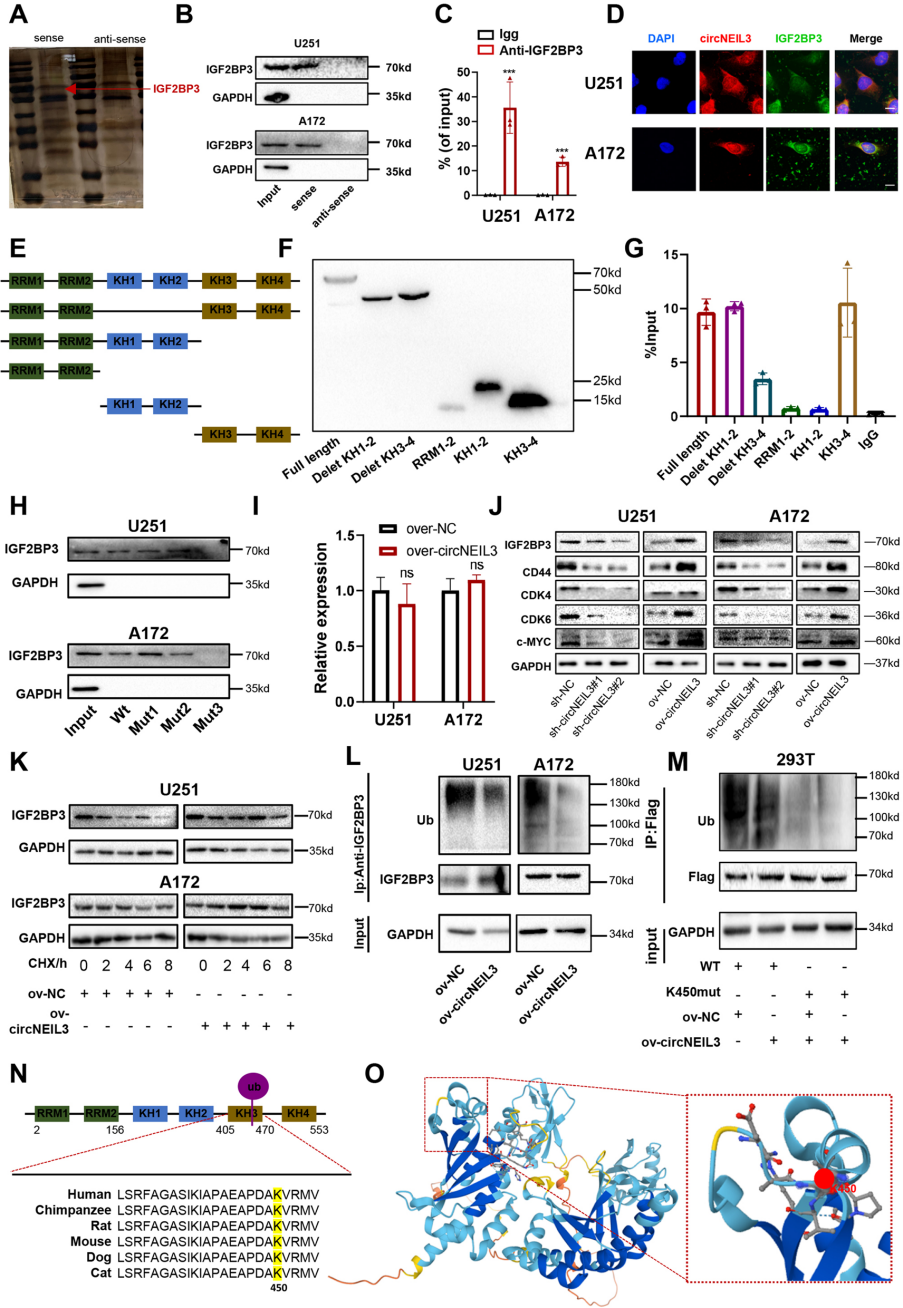
CircNEIL3 physically interacts with IGF2BP3 and inhibits its ubiquitination. **A** Different protein bands detected by silver stain for mass spectrometry of the circNEIL3-protein complex pulled down by circNEIL3 junction sense or anti-sense in U251 cells, the arrow points to IGF2BP3 band. **B** RNA pull down and western blot assays showing the interaction between IGF2BP3 with circNEIL3 in U251 and A172 cells. GAPDH was used as a negative control. **C** RIP and qRT-PCR assays showing the interaction between IGF2BP3 with circNEIL3 in GBM cells, using Igg and IGF2BP3 antibodies, *n* = 3. **D** IF-Fish assay showing co-localization of circNEIL3 (red) with IGFB2P3 proteins (green) in U251 and A172 cells. Scale bar, 25 μm. **E** Schematic structures of IGF2BP3 proteins and five mutants (Delet KH1–2, Delet KH3–4, RRM 1–2, KH1–2, KH3–4). **F** Western blot assay showing the full length of IGF2BP3 and its mutants. **G** RIP and qRT-PCR assays showed that the KH3–4 fragment of IGF2BP3 was the region responsible for circNEIL3 binding. **H** RNA pull-down and western blot assays showed that the interaction site on circNEIL3 with IGF2BP3. **I** The mRNA expression of IGF2BP3 was detected by qRT-PCR in GBM cells transfected with ov-NC or ov-circNEIL3, *n* = 3. **J** Western blot assays showing the protein levels of IGF2BP3 and downstream genes, including CD44, CDK4, CDK6 and c-MYC in GBM cells transfected with si-NC or si-circNEIL3, and ov-NC or ov-circNEIL3. **K** Western blot assays showing the protein levels of IGF2BP3 in GBM cells transfected with ov-NC or ov-circNEIL3, treated with 20 μg/ml CHX for the indicated periods of time. **L** Co-IP assay showing the ubiquitination modification level of IGF2BP3 in GBM cells transfected with ov-NC or ov-circNEIL3. Ub ubiquitin. **M** Co-IP assay showing the ubiquitination modification level of IGF2BP3 in GBM cells co-transfected with ov-NC or ov-circNEIL3 and vetors expressing FLAG-tagged WT or IGF2BP3 mutants (K450) as indicated. **N** Conservation ability of the K405 ub site on IGF2BP3 protein. **O** Crystal structure of IGF2BP3 proteins with K405. All data are presented as the means ± SD, ns, P > 0.05, ***p < 0.001

Studies have shown that cytoplasm-retained circRNAs usually perform their functions by acting as ceRNAs or scaffolds for RBPs [5, 6]. To explore the molecular mechanism of the circNEIL3-induced progression of GBM cells, we first performed RNA pull-down assays and subsequent mass spectrometry assays to explore potential proteins binding to circNEIL3. A total of 50 proteins interacting with circNEIL3 were identified, which did not include AGO2, excluding the possibility that circNEIL3 functions as a ceRNA (Additional file Table S2). We then intersected our mass spectrometry data with the RBPs predicted in the CircInteractome database. We found that circNEIL3 bound to IGF2BP3 protein (Fig. 4A, Fig. S4A). Then, we further confirmed the interaction between circNEIL3 and IGF2BP3 by RNA pull-down and RIP-qPCR assays (Fig. 4B, C). We also performed RNA FISH-immunofluorescence (FISH-IF) analysis and found that circNEIL3 colocalized with IGF2BP3 in the cytoplasm (Fig. 4D). IGF2BP3 consists of 2 RNA recognition motifs (RRMs) and 4 K homology (KH) domains; therefore, we established six FLAG-tagged vectors, which were confirmed by Western blot, to test which domain interacts with circNEIL3 (Fig. 4E, F). The RIP-qPCR assay showed that circNEIL3 mostly bound to the region of KH3–4, suggesting that the KH3–4 di-domain is responsible for recruiting circNEIL3 (Fig. 4G). We then searched for the motif necessary for IGF2BP3 recruitment in circNEIL3. Moreover, to identify the sequence in circNEIL3 to which IGF2BP3 binds, we used the CircInteractome database and found that two sequences may bind to IGF2BP3 (Fig. S4B, the yellow filling sequences). Interestingly, a study by Markus et al. [26] showed that the sequence CAUH (H = A, U or C) was the only one that could be recognized by IGF2BP3. Therefore, we reviewed the sequence found by the CircInteractome database and found three possible binding sites (Fig. S4C red font sequences). Then, we designed three mutant sites and finally observed that IGF2BP3 binds to the third site of circNEIL3 (Fig. 4H, Fig. S4D red font sequences). These results suggested that circNEIL3 physically interacts with IGF2BP3 in the cytoplasm.

We found that the expression level of IGF2BP3 increased with tumour grade, and patients with high expression had a worse prognosis than those with low expression in The Cancer Genome Atlas (TCGA) and Chinese Glioma Genome Atlas (CGGA) datasets (Fig. S5A- D). In addition, with increasing IGF2BP3 expression, the cancer-promoting signalling pathways were significantly enriched in the TCGA and CGGA datasets, as estimated via the ssGSEA algorithm (Fig. S5E, F), which is consistent with the enrichment analysis of circNEIL3 (Fig. S1C). Moreover, similar to circNEIL3 enrichment analysis, the GSEA results also showed that classical pathways involved in tumour pathogenesis were significantly enriched in the high IGF2BP3 expression group in the CGGA and TCGA datasets (Fig. S5G, H). Recently, Dixit Deobrat et al. [27] stated that IGF2BP3 played key roles in glioblastoma maintenance, promoting tumour heterogeneity, which highlights its role in the malignant progression of glioma. Furthermore, we found that circNEIL3 did not significantly change the mRNA level of IGF2BP3 (Fig. 4I) but significantly promoted its protein expression and as well as that of its downstream targets, including CDK4/6, CD44 and c-MYC (Fig. 4J), which are involved in the biological functions of cell proliferation, invasion and migration [28–30]. Rescue assays showed that IGF2BP3 knockout compensated for the increased proliferation, invasion and migration capacity, as well as upregulation of its downstream targets, caused by exogenous overexpression of circNEIL3 (Fig. S6A-E), indicating that circNEIL3 may regulate the malignant progression of glioma by destabilizing IGF2BP3 proteins.

Our further investigation showed that circNEIL3 overexpression enhanced the protein expression levels of IGF2BP3, extended the half-life of IGF2BP3(Fig. 4K), indicating that circNEIL3 regulated the stability of IGF2BP3 protein via proteasomal activity. Consistently, circNEIL3 overexpression decreased the ubiquitination of IGF2BP3 (Fig. 4L). We then predicted IGF2BP3 functional ubiquitylation sites via the Ubibrowser database (http://ubibrowser.ncpsb.org.cn/). Only one ubiquitinated lysine (K) residue (K450), which is located in the KH3 domain of IGF2BP3, was identified (Fig. 4N, Fig. S7A). Furtherly, we used uniport database (https://www.uniprot.org/) to predict that the K450 site of IGF2BP3 was highly conserved in six species (Fig. 4N). Moreover, we predicted the structure of IGF2BP3 through the Swiss Model Online website (https://swissmodel.expasy.org/) and visualized the K450 ubiquitination site (Fig. 4O). We then mutated the predictive site from lysine (K) to arginine (R) to confirm its role as a ubiquitination target. Immunoprecipitation (IP) results showed that the mutation of K450 significantly reduced the ubiquitination level of IGF2BP3 compared to that of WT IGF2BP3, and the enhanced ubiquitination caused by overexpression of cirNEIL3 was also inhibited in cells expressing the mutant (Fig. 4M), highlighting K450 as the major ubiquitination site of IGF2BP3. These results demonstrated that circNEIL3 enhanced the stability of IGF2BP3 protein by inhibiting ubiquitin/proteasome-dependent degradation, thereby promoting malignant progression of glioma.

### CircNEIL3 stabilizes IGF2BP3 protein by preventing HECTD4-mediated ubiquitination

**Fig. 5:**
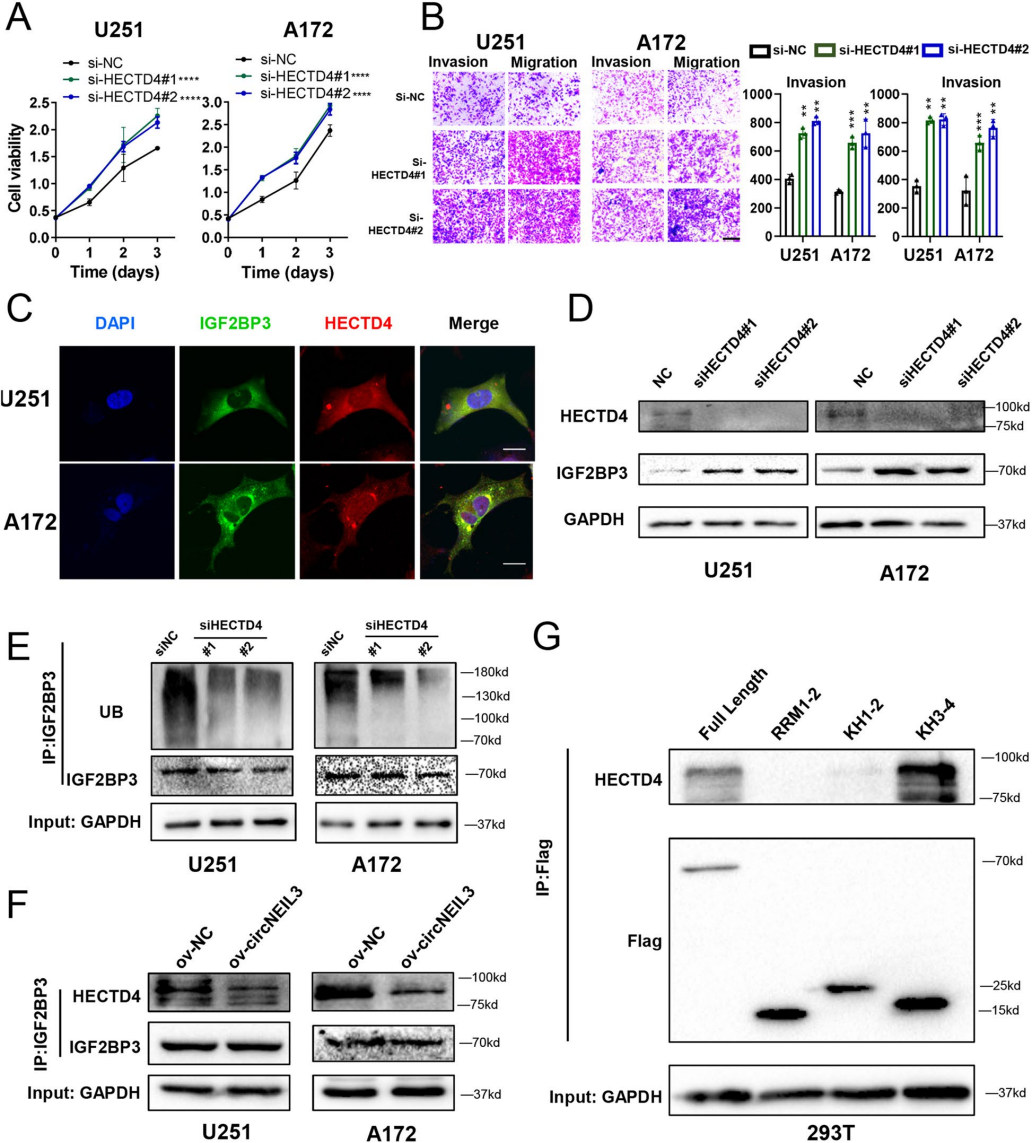
CircNEIL3 blocked the binding between IGF2BP3 and HECTD4. **A** CCk8 assays showing the proliferation ability of GBM cells transfected with si-NC or si-HECTD4. n = 3. **b**. Representative transwell migration and invasion assays showing the migration and invasion ability of GBM cells transfected with si-NC or si-HECTD4. Quantification histogram represented relative cell numbers, n = 3, scale bar, 200 μm. **C** IF assays showing the co-localization of IGF2BP3 (green) with HECTD4 (red) in GBM cells. Scale bar, 25 μm. **D** Western blot assays showing the expression of HECTD4 and IGF2BP3 in GBM cells transfected with si-NC or si-HECTD4. **E** Co-IP assays showing the ub modification level in GBM cells transfected with si-NC or si-HECTD4. **F** Co-IP assays showing the binding between HECTD4 with IGF2BP3 proteins in GBM cells transfected with ov-NC or ov-circNEIL3. **G** Co-IP and western blot assays showing the interaction between HECTD4 and different IGF2BP3 domain in in 293 T cells transfected with vectors expressing the Flag-tagged FL or the truncated mutants (RRM1–2, KH1–2, KH3–4), using Flag antibodies. All data are presented as the means ± SD, **P < 0.01, ***P < 0.001, ****P < 0.0001

The literature has shown that TRIM25 mediates the ubiquitination of IGF2BPs in NSCLC [31]. However, there is no report on the E3 ubiquitin ligase that mediates the ubiquitination of IGF2BP3 in glioma. Thus, we sought to identify the E3 ligase involved in proteasome-mediated degradation of IGF2BP3 in glioma. We performed co-immunoprecipitation (co-IP) experiments and mass spectrometry analysis and found that HECTD4, a protein belonging to the HECD family of E3 ubiquitin ligases that links polyubiquitin to the target protein to promote their ubiquitination [32, 33], could bind to IGF2BP3 (Fig. S7B, and Additional file Table S3). The expression level of HECTD4 decreased with increasing tumour grade, and patients with low expression had a worse prognosis than those with high expression in the TCGA datasets (Fig. S7C, D). Moreover, we validated that HECTD4 can inhibit GBM cell proliferation, invasion and migration in vitro (Fig. 5A, B, Fig. S7E, F). To confirm that HECTD4 could regulate the ubiquitination of IGF2BP3, we performed immunofluorescence (IF) experiments and confirmed that IGF2BP3 colocalized with HECTD4 in the cytoplasm (Fig. 5C). Furthermore, we found that the protein level of IGF2BP3 was dramatically increased (Fig. 5D), while its level of ubiquitination was obviously decreased, in HECTD4-knockdown GBM cells (Fig. 5E), indicating that HECTD4 acts as an E3 ubiquitin ligase to degrade IGF2BP3 via the ubiquitin–proteasome pathway in glioma.

Mass spectrometry analysis of circNEIL3 showed that circNEIL3 did not bind to HECTD4 (Additional file Table S2), but we found that overexpression of circNEIL3 can block the binding between IGF2BP3 and HECTD4 (Fig. 5F), which explain how circNEIL3 stabilizes IGF2BP3. To further confirm that circNEIL3 can inhibit the binding of IGF2BP3 to HECTD4, we performed a Co-IP assay and observed that HECTD4 bound to the KH3–4 domains of IGF2BP3, which is the same site where circNEIL3 interacts with IGF2BP3 (Fig. 5G, and Fig. 4G). These data suggested that circNEIL3 blocked the binding between IGF2BP3 and HECTD4 via steric hindrance, ultimately inhibiting the ubiquitination of IGF2BP3. Collectively, these data showed that circNEIL3 stabilizes IGF2BP3 protein by preventing HECTD4-mediated ubiquitination.

### CircNEIL3 facilitates macrophage infiltration in glioma

Recent evidence suggests that glioma cells can recruit immune cells to the TME, thereby maintaining glioma pathology and promoting malignant progression [12, 34]. Intriguingly, our ssGSEA enrichment results showed that compared to tumours with low circNEIL3 expression, tumours with high circNEIL3 expression were enriched not only in carcinogenic signalling pathways but also in immune-related pathways, such as inflammatory response and TGF-beta signalling (Fig. S1C). Likewise, patients in the TCGA and CGGA datasets with high expression of IGF2BP3 also presented consistent results (Fig. S5E, F), suggesting that circNEIL3 is involved in immune regulation. To explore the differences in the biological behaviours among samples with distinct circNEIL3 expression, we divided the glioma samples into high and low expression groups based on the median expression of circNEIL3. Differential expression analysis was performed using the Deseq2 package, and a total of 801 genes were differentially expressed in the high expression group compared with the low circNEIL3 expression group; 531 genes were upregulated and 270 genes were downregulated (fold change ≥2 and *P*-value< 0.05, Additional file Table S4). Furthermore, Metascape database [35] analysis revealed that the differentially upregulated genes were markedly enriched in the regulation of cell biological functions, stromal activation and immune-related pathways (Fig. S8A, Additional file Table S5). Enrichment analysis with PaGenBase [36] showed that these genes were almost completely specifically expressed in spleen, blood, bone marrow and other peripheral immune organs (Fig. S8B). We then stratified glioma samples from the TCGA datasets into high and low expression groups based on the median expression of IGF2BP3 and obtained similar results through differential expression and enrichment analysis (Fig. S8C, D, Additional file Tables S6, S7). GSEA also demonstrated that immune-related signatures, including inflammatory response, TGF beta signalling, protein secretion, and leukocyte migration, were highly enriched in circNEIL3-high samples compared with circNEIl3-low samples (Fig. S8E). GSEA of IGF2BP3 in TCGA glioma samples uncovered similar results (Fig. S8F), indicating that circNEIL3 may be involved in the regulation of TME cells.

**Fig. 6:**
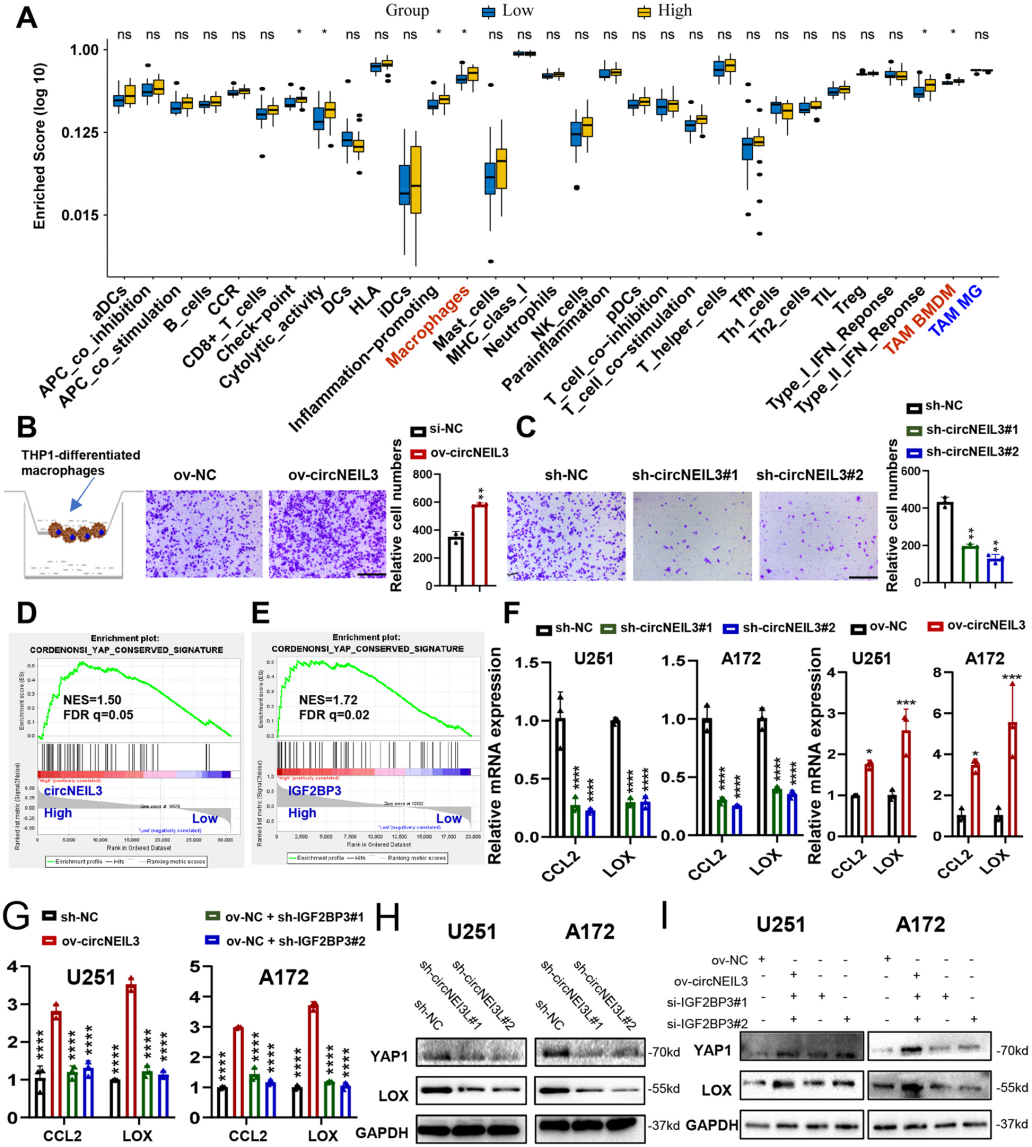
CircNEIL3 facilitates macrophages infiltration in glioma. **A** The abundance of each TME infiltrating cells and regulators in circNEIL3 high and low groups. The statistical *p*-value was calculated using the nonparametric Wilcoxon test. Representative transwell migration assays showed the chemotaxis ability of human THP1-differentiated macrophages by exposing them to conditioned medium (CM) from GBM cells transfected with **(B)** ov-NC or ov-circNEIL3, and **(C)** sh-NC or sh-circNEIL3. Quantification histogram represented relative cell numbers, n = 3, scale bar, 200um. GSEA of CONRDENONsi YAP Conserved signature showed that glioma samples with high **(D)** circNEIL3 in our local glioma dataset, and **(E)** IGF2BP3 expression in TCGA glioma dataset were enriched in the YAP1 signaling compared to glioma samples with low expression, respectively. NES, normalized enrichment score; FDR, false discovery rate. **F** qRT-PCR assays showing the relative mRNA expression of CCL2 and LOX in GBM cells transfected with (left) sh-NC or sh-circNEIL3, and (right) ov-NC or ov-circNEIL3, n = 3. **G** qRT-PCR assays showing the relative mRNA expression of CCL2 and LOX in GBM cells co-transfected with ov-NC, ov-circNEIL3 and sh-IGF2BP3 as indicated, n = 3, and the ov-circNEIL3 group is indicated as the control. Western blot assays showing the protein expression of YAP1 and LOX in GBM cells **(H)** transfected with sh-NC or sh-circNEIL3, and **(I)** co-transfected with ov-NC, ov-circNEIL3 and sh-IGF2BP3 as indicated. All data are presented as the means ± SD, ns, P > 0.05, *P < 0.05, **P < 0.01, ***P < 0.001, ****P < 0.0001

To further understand the exact role of circNEIL3 in the immunophenotype of glioma, we assessed glioma purity and stromal and immune scores using the ESTIMATE algorithm and analysed 31 gene sets representing different immune cell types, functions, and pathways via the gene set variation analysis (GSVA) algorithm (see Materials and Methods). As shown in Fig. 6A and Fig.S9, the circNEIL3 high expression group had greater cell infiltration into the TME, higher immune and stromal scores, and lower tumour purity than he circNEIL3 low expression group. Compared to gliomas with low circNEIL3 expression, gliomas with high circNEIL3 expression exhibited significantly increased macrophage infiltration, which constitute the most abundant cell population in the glioma TME. To identify specific macrophage populations associated with circNEIL3 expression, we analysed TAM MGs and TAM BMDMs in glioma samples using validated gene signatures [21]. The results showed that gliomas with high circNEIL3 expression exhibited significantly increased infiltration of TAM BMDMs (hereafter also referred to as macrophages), while the number of TAM MGs was decreased to some extent (Fig. 6A, Fig. S9). GSEA also showed that myeloid leukocyte migration and macrophage activation involved in immune response pathways were highly enriched in circNEIL3-high samples compared with circNEIl3-low samples (Fig. S8E). Similar to circNEIL3, high IGF2BP3 expression in glioma samples from the TCGA dataset showed similar results (Fig. S8F, Fig. S10A-D), suggesting that circNEIL3 is involved in the recruitment of macrophages. To further confirm the role of circNEIL3 in facilitating macrophage migration, we used the Transwell assay and found that conditioned medium (CM) from circNEIL3-overexpressing GBM cells significantly promoted THP1-differentiated macrophage migration compared to that of the NC group (Fig. 6B), while CM from circNEIL3-knockdown GBM cells showed the opposite results (Fig. 6C). Taken together, our results demonstrated that circNEIL3-overexpressing tumour cells could drive macrophage infiltration into the glioma microenvironment.

Next, we explored the potential regulatory mechanism by which circNEIl3 promotes macrophage recruitment. Recently, Chen et al. [11] found that PTEN deficiency in GBM increases macrophage infiltration by activating YAP1 signalling, which directly upregulates the expression of lysyl oxidase (LOX), a powerful macrophage chemoattractant. The infiltrated macrophages in turn secrete SPP1, which is a member of the largest group of proteins specifically expressed by macrophages [14], to support GBM survival. In addition, C-C motif chemokine ligand 2 (CCL2), another powerful macrophage-secreted chemokine, is also a target of YAP1 [37]. Our GSEA results demonstrated that the YAP1 signalling gene signature was highly enriched in circNEIL3-high samples compared with circNEIL3-low samples (Fig. 6D). Further ssGSEA revealed that compared to tumours with low circNEIL3 expression, tumours with high circNEIL3 expression exhibited higher levels of CCL2 and LOX (Fig. S10E). Assessment of IGF2BP3 in TCGA glioma samples also showed the same results (Fig. 6E, Fig. S10E). Our qRT-PCR assays validated the results that circNEIL3 promoted the expression of CCL2 and LOX (Fig. 6F), and this enhanced expression induced by circNEIL3 overexpression could be rescued by IGF2BP3 knockdown (Fig. 6G). Finally, we confirmed that circNEIL3 could increase the protein expression of YAP1 and LOX in GBM cells (Fig. 6H). The increased expression caused by circNEIL3 overexpression also could be abrogated by IGF2BP3 knockdown (Fig. 6I). In summary, these results demonstrated that circNEIL3-overexpressing GBM cells might drive macrophage infiltration into the tumour-associated microenvironment by activating YAP1 signalling.

### CircNEIL3 can be packaged into exosomes by hnRNPA2B1

Past studies indicated that circRNAs could be packaged into exosomes and play an important role in the progression of tumours [38, 39]. Then, to investigate whether circNEIL3 can be loaded into exosomes, we collected exosomes from the supernatants of cultured GBM cells, which exhibited similar typical cup-shaped morphology, size, and number (Fig. S11A, B), and further confirmed their identity by detection of the exosome markers TSG101 and CD9 (Fig. S11C), indicating that we successfully isolated exosomes from GBM cells. Furthermore, we found that the expression of circNEIL3 was downregulated in cells treated a pharmacological inhibitor of neutral sphingomyelinase-2 (nSMase) GW4869, which blocks exosome formation (Fig. S11D), thus confirming the existence of circNEIl3 in exosomes. Finally, we showed that circNEIL3 overexpression in GBM cells led to increased levels of circNEIL3 expression in exosomes, while circNEIL3 knockdown in the same cell line produced the opposite results (Fig. S11E). Altogether, these results show that circNEIL3 could be packaged into exosomes.

**Fig. 7:**
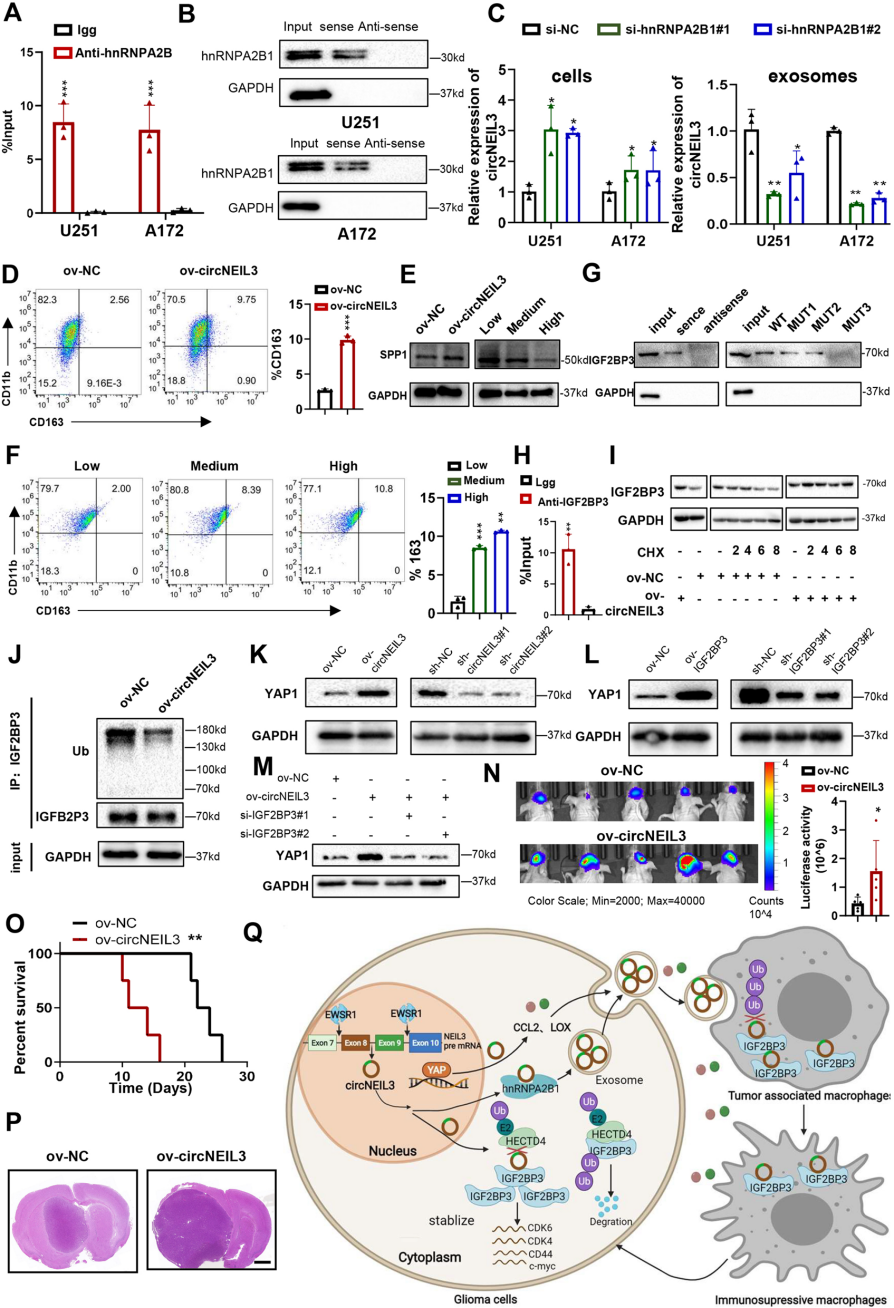
Exosomes could deliver circNEIL3 to TAMs, thereby enabling them to acquire angiogenic and immunosuppressive properties. **A** RIP and qRT-PCR assays showing the interaction brtween circNEIL3 and hnRNPA2B1, using Igg and hnRNPA2B1 antibodies, n = 3. **B** RNA pulldown and western blot assays showing the interaction brtween circNEIL3 and hnRNPA2B1. qRT-PCR assay showing the relative expression in (left) GBM cells transfected with si-NC or si-hnRNPA2B1, and (right) exosomes collected from GBM cells transfected with si-NC or si-hnRNPA2B1, n = 3. **D** Representative flow cytometry assay showing the proportion of CD11b + CD163+ in THP1 differentiated macrophages transfected with ov-NC or ov-circNEIL3. Quantification histogram represented the proportion of CD11b + CD163+ in THP1 differentiated macrophages, n = 3. **E** Western blot assays showing the expression of SPP1 in THP1 differentiated macrophages (Left) transfected with ov-NC or ov-circNEIL3, and (Right) treated with exosomes collected from GBM cells transfected with si-circNEIL3, blank or ov-circNEIL3. **F** Representative flow cytometry assay showing the proportion of CD11b + CD163+ in THP1 differentiated macrophages treated with exosomes collected from GBM cells transfected with si-circNEIL3, blank or ov-circNEIL3. Quantification histogram represented the proportion of CD11b + CD163+ in THP1 differentiated macrophages, n = 3. **G** RNA pulldown and western blot assays showing the interaction between circNEIL3 and IGF2BP3 in THP1 differentiated macrophages in the indicated group. **H** RIP and qRT-PCR assays showing the interaction between circNEIL3 and hnRNPA2B1, using Igg and IGF2BP3 antibodies, n = 3. **I** Western blot assays showing the protein levels of IGF2BP3 in THP1 differentiated macrophages transfected with ov-NC or ov-circNEIL3, treated with 20 μg/ml CHX for the indicated periods of time. **J** Co-IP assay showing the ubiquitination modification level of IGF2BP3 in THP1 differentiated macrophages transfected with ov-NC or ov-circNEIL3. Western blot assays showing the protein levels of YAP1 in THP1 differentiated macrophages transfected with (**K**) left, ov-NC or ov-circNEIL3, and right, sh-NC or sh-circNEIL3 as indicated, and (**L**) left, ov-NC or ov-IGF2BP3, and right, sh-NC or sh-IGF2BP3 as indicated. **M** Western blot assays showing the YAP1 protein expression in THP1-differentiated macrophages co-transfected with ov-NC or ov-circNEIL3 and si-IGF2BP3 as indicated. **N** Left, representative bioluminescent image showing the tumor size for animals in different groups in the indicated time. *n* = 5 for each group. Right, statistical analysis of bioluminescent tracking plots. **O** Kaplan-Meier survival curves for animals in different groups, n = 5 for each group. **P** Represented H&E staining images for a subgroup of animal sacrificed simultaneously in each group, n = 5 for each group, scale bar, 10 μm. **Q** Proposed working model of circNEIL3 functions in tumorgenesis and malignant progression of glioma. All data are presented as the means ± SD, *p < 0.05, **p < 0.01, ***p < 0.001, ****p < 0.0001

We next investigated the mechanism by which circNEIL3 is packaged into exosomes. We first analysed the mass spectrometry data of the circNEIL3 pull-down experiments and found that circNEIL3 could bind to hnRNPA2B1, which can transport various RNAs into exosomes [40, 41] (Fig. S11F, Additional file Table S2). We then confirmed the interaction between circNEIL3 and hnRNPA2B1 by RIP and RNA pull-down assays in GBM cells (Fig. 7A, B). Moreover, we found that circNEIL3 expression was upregulated in cells and downregulated in exosomes in hnRNPA2B1-knockdown GBM cells (Fig. 7C). In summary, these results indicated that circNEIL3 could be packaged into exosomes by hnRNPA2B1.

### Exosomes deliver circNEIL3 to TAMs, thereby enabling them to acquire immunosuppressive properties by stabilizing IGF2BP3

Exosomes play a vital role in the communication between cancer cells and TAMs, and we found that compared to tumours with low circNEIL3 expression, glioma samples with high circNEIL3 expression exhibited significantly increased expression of a panel of macrophage-derived immunosuppressive genes [12, 13], including CD163, TGFB1, IL1RA, IL10, ARG1 and PD-L1 (Fig. S10E). Our qRT-PCR analysis data also confirmed that circNEIL3 overexpression could significantly upregulate the expression of these genes in THP1-differentiated macrophages (Fig. S11G). Flow cytometry results further demonstrated that circNEIL3 overexpression significantly upregulated the macrophage activation marker CD163 (Fig. 7D). We observed that circNEIL3 overexpression significantly upregulated the protein expression of SPP1, which sustains glioma cell survival and stimulates angiogenesis [11]. Therefore, we hypothesized that exosomes could deliver circNEIL3 to macrophages to regulate the immunosuppressive phenotype of macrophages. First, we performed IF to confirm that exosomes can be taken up by macrophages (Fig. S11H). We then collected exosomes from GBM cells with normal expression, overexpression, or knockdown of circNEIL3 and divided them into high, medium and low expression groups before using them to treat THP1-differentiated macrophages (Fig. S11I). We found that macrophage activation markers, immunosuppressive molecules, and SPP1 were significantly upregulated with the increased expression of circNEIL3 in exosomes (Figs. 7E, F, Fig. S11J). Overall, these results suggested that exosomes could deliver circNEIL3 to TAMs, thereby enabling them to acquire angiogenic and immunosuppressive properties.

To clarify the mechanisms by which circNEIL3 mediates macrophage immunosuppressive properties, we performed RNA pull-down and RIP assays and found that circNEIL3 bound to IGF2BP3 at the same site in THP1-differentiated macrophages as in tumour cells (Fig. 7G, H). Moreover, as observed in tumour cells, circNEIL3 increased IGF2BP3 protein expression and inhibited IGF2BP3 ubiquitination in differentiated THP1 macrophages (Fig. 7I, J). A recent study reported that IGF2BP2, a member of the IGF2BP family, could promote macrophage polarization [42]. Meanwhile, IGF2BPs have similar 4 KH domains and 2 RRMs and play a similar role in various types of cells [31, 43]. Therefore, we hypothesized that, like that in GBM cells, IGF2BP3 can act as the downstream effector of circNEIL3 in macrophages and mediate the polarization of macrophage toward an immunosuppressive phenotype. Similar to our observations with circNEIL3, the panel of macrophage-derived immunosuppressive genes was dramatically upregulated in IGF2BP3 high expression gliomas compared with IGF2BP2 low expression gliomas in the TCGA dataset (Fig. S10E). Data from the qRT-PCR and flow cytometry assays confirmed that IGF2BP3 overexpression could significantly upregulate the expression of these genes in THP1-differentiated macrophages (Fig. S11K, M). Furthermore, the enhanced expression of these genes induced by circNEIL3 overexpression could be abolished by IGFB2P3 knockdown (Fig. S11L, N). Several studies have found that high YAP expression in macrophages can also promote their polarization toward the M2 phenotype [44, 45]. Thus, we hypothesized that circNEIL3 might also promote polarization of the immunosuppressive phenotype in macrophages by stabilizing IGF2BP3 protein to promote the expression of YAP1. Our western blot assays validated the results that overexpression of both circNEIL3 and IGF2BP3 enhances YAP1 protein expression in THP1-differentiated macrophages, while knocking down of them showed the opposite result (Fig. 7K, L). Furthermore, the enhanced protein expression of YAP1 induced by circNEIL3 overexpression could be abolished by IGFB2P3 knockdown (Fig. 7M), suggesting that circNEIL3 enhanced YAP1 expression by stabilizing the IGF2BP3 protein, which in turn promoted immunosuppressive phenotypic polarization in macrophages.

To further validate the effect of circNEIL3 on macrophage immunosuppressive polarization in vivo, macrophages overexpressing cicrNEIL3 or negative control vector were co-implanted with glioma cells into the brains of nude mice in situ. We found that compared to the NC group, the circNEIL3 overexpression group displayed elevated tumour growth and prolonged survival of tumour-bearing mice (Fig. 7N-P). Moreover, to further demonstrate that circNEIL3 exerts its function through the IGF2BP3-YAP1 axis, we then performed immunofluorescence staining assay in our local clinical glioma patient’s tissues. As shown in Fig. S11O, the expression of IGF2BP3, YAP1, CD68 (human macrophage marker) was enhanced in circNEIL3-high glioma tissues compared to circNEIL3-low tissues. Overall, these results indicated that IGF2BP3 can act as the downstream effector of circNEIL3 in macrophages and enable them to acquire immunosuppressive properties, in turn promoting glioma progression.

## Discussion

Emerging evidence has indicated that circRNAs, novel noncoding RNAs, play a critical role in various types of tumours [7, 8]. In this study, we first demonstrated that circNEIL3, which is derived from NEIL3, is frequently upregulated in glioma tissues, and its expression increases with increasing glioma grade. CircNEIL3 can be cyclized by the known RBP EWSR1, which is more active in hypoxic environments [46–48], resulting in higher expression of circNEIL3 under hypoxia. Functionally, we confirmed that circNEIL3 promoted tumorigenesis and progression of glioma in vitro and in vivo. Mechanistically, circNEIL3 stabilizes IGF2BP3 protein, a known oncogenic protein, by preventing its ubiquitination mediated by HECTD4. Moreover, circNEIL3-overexpressing glioma cells drive macrophage infiltration into the TME by activating YAP1 signalling. Finally, circNEIL3 could be packaged into exosomes by hnRNPA2B1 and transmitted to infiltrating glioma-associated macrophages, thereby enabling them to acquire immunosuppressive properties by stabilizing IGF2BP3, which in turn promotes glioma progression (Fig. 7Q). One unique attribute of our research is that we demonstrate that the novel circRNA, circNEIL3, can impact glioma progression via cancer cells and their complex network of interactions with macrophages, a key component of the TME. Interestingly, in both types of cells, circNEIL3 inhibited the ubiquitination of IGFB2P3 in the same way, highlighting that circNEIL3 may be a potential prognostic biomarker and therapeutic target in glioma.

The most important mechanism of cyclization of circRNAs is dependent on RBPs located on introns upstream and downstream of the coding region [23, 49]. Various RBPs induce the upregulation of tumour-associated circRNAs [23, 50], such as eukaryotic translation initiation factor 4A3 (EIF4A3), which upregulates circMMP9 in GBM [51]. We showed that EWSR1 could bind to upstream and downstream introns of circNEIL3, promoting the cyclization of circNEIL3 (Fig. 3).

As a common feature of solid tumours, hypoxia is associated with poor prognosis and tumour aggressiveness. Recent studies show that hypoxia can upregulate various circRNAs in different cancers [52–54]. However, the mechanism by which hypoxia upregulates circRNAs in tumours is not clear. In our study, we found that EWSR1 is more active under hypoxia, inducing higher expression of circNEIL3 (Fig. 3G-H). Our findings can explain how hypoxia induces upregulation of circRNAs in various types of tumours.

IGF2BP3 belongs to a novel family of RBPs (IGF2BPs), which were identified based on their ability to bind to various downstream mRNAs, such as c-myc, CDK4, and CDK6, and regulate their stability [43, 55–58]. Recently, Dixit Deobrat et al. [27] reported that IGF2BP3 played key roles in glioblastoma maintenance and promoting tumour heterogeneity, highlighting its role in the malignant progression of glioma. However, the mechanism of IGF2BP3 upregulation in glioma remains unclear. Recent research indicated that IGF2BP ubiquitination can be facilitated by circNDUFB2 as a scaffold to enhance the binding between TRIM25 and IGF2BPs in lung cancer [31]. Unlike the findings in lung cancer, we first demonstrated HECTD4 as an E3 ubiquitin ligase that can induce ubiquitination of IGF2BP3 in glioma. Meanwhile, we observed that circNEIL3 can bind to IGFB2P3 to block the interaction between IGF2BP3 and HECTD4 and induce the upregulation of IGF2BP3 in glioma (Fig. 5). Our data provide a novel mechanism for IGF2BP3 upregulation in GBM and new evidence that circRNAs mediate protein ubiquitination.

TAMs are the most abundant type of infiltrating immune cells in the glioma TME and have been revealed as critical cell components for gliomagenesis and malignant progression [12, 15]. Recent studies suggest that noncoding RNAs, such as miRNAs and lncRNAs, can be transported into TAMs from glioma cells via exosomes and mediate the immunosuppressive phenotype polarization of TAMs [17]. However, little is known about the regulatory mechanism of exosomal circRNAs and TAMs in glioma. Our study offers another significant finding that glioma-derived exosomes could deliver circNEIL3 to TAMs, thereby enabling them to acquire angiogenic and immunosuppressive properties, in turn promoting glioma progression (Figs. 6 and 7). IGF2BP3, as an RBP, can stabilize various mRNA targets, such as c-myc, which can mediate immune suppression of macrophages [59]. However, there is no direct evidence on whether IGF2BP3 can regulate macrophage activation and immunosuppressive phenotypic transformation. Our data demonstrated for the first time that IGF2BP3 could facilitate macrophages in the glioma TME and then reshape them to acquire an immunosuppressive phenotype. Moreover, circNEIL3 can promote this process by inhibiting the ubiquitination of IGF2BP3. However, how IGF2BP3 induces this immunosuppressive polarization remains largely unknown, and further studies will help to elucidate the effects of IGF2BP3 on GBM cells and its immune microenvironment, thereby providing a basis for the diagnosis and treatment of glioma.

## Conclusion

In conclusion, we identified a novel circular RNA, circNEIL3, that was upregulated in human glioma tissues. The expression of circNEIL3, which could be regulated by EWSR1, increased with the increasing glioma grade. Functionally, we confirmed that circNEIL3 promoted tumorigenesis and the progression of glioma in vitro and in vivo. Mechanistically, circNEIL3 stabilizes IGF2BP3, a known oncogenic protein, by preventing HECTD4-mediated ubiquitination. Moreover, circNEIL3-overexpressing glioma cells could drive macrophage infiltration into the TME. Finally, circNEIL3 could be packaged into exosomes by hnRNPA2B1 and transmitted to infiltrating TAMs, thereby enabling them to acquire immunosuppressive properties by stabilizing IGF2BP3, in turn promoting gliomagenesis and malignant progression. Our findings suggest that circNEIL3 could be a novel prognostic biomarker and promising therapeutic target for glioma.

## Supplementary Information

**Additional file 1: Table S1**. Genes positively correlated with circNEIL3. **Table S2**. Interacting proteins identified by circNEIL3 pulldown assays. **Table S3**. Interacting proteins identified by IGF2BP3 Co-IP assays. **Table S4**. Genes differentially expressed in circNEIL3 high group. Compared with low group. **Table S5**. Enriched terms across upregulated genes in circNEIL3 high group. **Table S6**. Genes differentially expressed in IGF2BP3 high group. Compared with low group in TCGA glioma cohort. **Table S7**. Enriched terms across upregulated genes in IGF2BP3 high group. **Table S8**. The detailed clinicopathological characteristics of glioma patients in our local cohort. **Table S9**. The detailed qRT-PCR primers used in this study. **Table S10**. The detailed siRNA sequences used in this study. Table S11. The detailed antibody information used in this study.

**Additional file 2: Figure S1**. Functional analysis of positive genes correlated with circNEIL3 in our local glioma tissues. A KEGG pathway analysis of the positive regulated genes with circNEIL3 in our local glioma dataset. B GSEA analysis revealed that genes with high circNEIL3 expression group were enriched for pathways of malignant tumors in our local glioma dataset. C GSVA enrichment analysis showing the activation status of biological pathways in the circNEIL3-high and circNEIL3-low groups. The heatmap was used to visualize these biological processes. Yellow represents activated pathways, black represents moderately activated pathways, and blue represents inhibited pathways. **Figure S2**. circNEIL3 promotes gliomagenesis in vitro. qRT-PCR assays showing the expression of (left) circNEIL3 and mRNA NEIl3(right) in GBM cells and GSCs transfected with (A) si-NC or si-circNEIL3, and (B) ov-NC or ov-circNEIL3, *n* = 3. Representative EDU assay images showing the proliferation ability of GBM cells transfected with (C) si-NC or si-circNEIL3, and (D) ov-NC or ov-circNEIL3. Quantification histogram represented relative cell numbers, n = 3, scale bar, 200um. Representative colony formation assays showing the proliferation ability of GBM cells transfected with (E) si-NC or si-circNEIL3, and (F) ov-NC or ov-circNEIL3. Quantification histogram represented relative cell numbers, n = 3. G Representative images of tumor spheres formation assays showing the tumor spheres formation ability of GSCs transfected with (G) si-NC or si-circNEIL3, and (H) ov-NC or ov-circNEIL3. Quantification histogram represented average sphere dimeter, *n* = 3, scale bar, 200um. limiting dilution assay showing the self-renewal ability of GSCs transfected with (I) si-NC or si-circNEIL3, and (J) ov-NC or ov-circNEIL3. K Represented H&E staining images for a subgroup of animal sacrificed simultaneously in each group, *n* = 5 for each group, scale bar, 10 μm. All data are presented as the means ± SD, *P < 0.01, **P < 0.01, ***P < 0.001, ****P < 0.0001. **Figure S3**. EWSR1 promotes the biogenesis of circNEIL3. A The binding sites of EWSR1 were predicted in the upstream and downstream region of NEIL3 pre-mRNA using circinteratome database. B The binding motif of EWSR1 were predicted in the upstream and downstream region of NEIL3 pre-mRNA using starbase database. C Western blot assay showing the expression of EWSR1 in GBM cells transfected with si-NC or siEWSR1. D The relative expression of EWSR1 in TCGA, CGGA and Rembrandt datasets. E Kaplan–Meier survival curves for EWSR1 in TCGA, CGGA and Rembrandt datasets. F Representative EDU assays showing the proliferation ability of GBM cells transfected with si-NC or si-EWSR1. Quantification histogram represented relative cell numbers, *n* = 3, scale bar, 200um. All data are presented as the means ± SD, *P < 0.01, **P < 0.01, ***P < 0.001, ****P < 0.0001. **Figure S4**. CircNEIL3 physically interacts with IGF2BP3. A Venn diagram showing the overlapping of interacted proteins with circNEIL3 between our mass spectrometric data and proteins predicted in CircInteractome database. B The sequences that may bind to IGF2BP3 protein predicted in CircInteractome database. C and D Sequences of circNEIL3 and circNEIL3 mutant as indicated. **Figure S5**. IGF2BP3 is correlated with carcinogenic activation pathways in TCGA and CGGA datasets. A The GEPIA database showing the relative expression of IGF2BP3 in TCGA glioma tissues compared with GETx normal brain. B Kaplan–Meier survival curves for IGF2BP3 in TCGA glioma dataset. C Relative expression of IGF2BP3 in CGGA glioma dataset. B Kaplan–Meier survival curves for IGF2BP3 in CGGA glioma dataset. GSVA enrichment analysis showing the activation status of biological pathways in the IGF2BP3-high and IGF2BP3-low groups in (E) TCGA glioma dataset, and (F) CGGA glioma dataset. A heatmap was used to visualize these biological processes. Yellow represents activated pathways, black represents moderately activated pathways, and blue represents inhibited pathways. GSEA analysis revealed that genes with high IGF2BP3 expression group were enriched for pathways of malignant tumors in (G) TCGA glioma dataset, and (H) CGGA glioma dataset. **Figure S6**. circNEIL3 regulated the malignant progression of glioma via destabilizing IGF2BP3 proteins. (A) CCK8 assays, and (B) Representative colony formation assays, and (C) Representative EDU assays showing the proliferation ability of GBM cells co-transfected with ov-NC or ov-circNEIL3 and si-IGF2BP3 as indicated. Quantification histogram represented relative cell numbers, *n* = 3, scale bar, 200 μm. D Representative transwell migration and invasion assays showing the migration and invasion ability of GBM cells co-transfected with ov-NC or ov-circNEIL3 and si-IGF2BP3 as indicated. Quantification histogram represented relative cell numbers, n = 3, scale bar, 200um. E Western blot assays showing the expression of CD44, CDK4, CDK6 and c-MYC in GBM cells co-transfected with ov-NC or ov-circNEIL3 and si-IGF2BP3 as indicated. All data are presented as the means ± SD, and the ov-circNEIL3 group is indicated as the control, **P < 0.01, ***P < 0.001, ****P < 0.0001. Figure S7. circNEIL3 stabilizes IGF2BP3 protein by preventing HECTD4-mediated ubiquitination. A The ubiquitination site of IGF2BP3 predicted via Ubibrowsers database. B Co-IP assays were performed to identify the IGF2BP3-protein complex by IGF2BP3 antibody in GBM cells. C The GEPIA database showing the relative expression of HECTD4 in glioma tissues compared with GETx normal brain tissues. D Kaplan–Meier survival curves for IGF2BP3 in TCGA glioma dataset. E Colony formation assays showing the proliferation ability of GBM cells transfected with si-NC or si-HECTD4. F Representative EDU assays showing the proliferation ability of GBM cells transfected with si-NC or si-HECTD4. Quantification histogram represented relative cell numbers, n = 3, scale bar, 200um. All data are presented as the means ± SD, and the ov-circNEIL3 group is indicated as the control, *P < 0.05, **P < 0.01. **Figure S8**. The genes that upregulated in circNEIL3 and IGF2BP3 high expression group were enriched in immune response- and stromal-related pathways. Bar graph of (A) enriched terms, colored by *p*-values, and (B) Summary of enrichment analysis in PaGenBase across upregulated genes in circNEIL3 high group in our local glioma samples. Bar graph of (C) enriched terms, colored by p-values, and (D) Summary of enrichment analysis in PaGenBase database across upregulated genes in IGF2BP3 high group in TCGA glioma samples. GSEA analysis revealed that genes with (E) high circNEIL3 expression group in our local glioma samples, and (F) high IGF2BP3 expression in TCGA glioma samples were enriched for immune response related pathways. **Figure S9**. CircNEIL3 is correlated with immunophenotypes and TME landscapes. GSVA enrichment analysis showing the enrichment scores of immune cell types and immune-related function related gene sets in the circNEIL3-high and circNEIL3-low groups. A heatmap was used to visualize these immune characteristics between the circNEIL3 high and low expression groups; yellow represents a high enrichment level, black represents a median enrichment level, and blue represents a low enrichment level. **Figure S10**. IGF2BP3 is correlated with immunophenotypes and TME landscapes. GSVA enrichment analysis showing the enrichment scores of immune cell types and immune-related function related gene sets in the IGF2BP3-high and IGF2BP3-low groups in (A) TCGA glioma dataset, and (B) CGGA glioma dataset. A heatmap was used to visualize these immune characteristics between the IGF2BP3 high and low expression groups; yellow represents a high enrichment level, black represents a median enrichment level, and blue represents a low enrichment level. The abundance of immune/stromal score, each TME infiltrating cells and regulators in IGF2BP3 high and low groups in (C) TCGA dataset, and (D) CGGA dataset. E GSVA enrichment analysis showing the immunosuppressive genes in the (left) circNEIL3-high and circNEIL3-low groups in our local glioma samples, and (right) IGF2BP3-high and IGF2BP3-low groups in TCGA glioma samples. The statistical p-value was calculated using the nonparametric Wilcoxon test, ns, *P* > 0.05, *P < 0.05, **P < 0.01, ***P < 0.001, ****P < 0.0001. **Figure S11**. Exosomes deliver circNEIL3 to TAMs, thereby enabling them to acquire immunosuppressive properties via stabilizing IGF2BP3. A Transmission electron microscopy detected the exosomes isolated from supernatants of GBM cells via ultracentrifugation, scale bar, 200 nm. B NanoSight particle tracking analysis of the size distributions and number of exosomes. C Western blot assay showing the exosomes markers (CD9, Calnexin and TSG101) in exosomes isolated from supernatants of GBM cells via ultracentrifugation and corresponding GBM cells. qRT-PCR assay showing the expression of circNEIL3 in exosomes isolated from supernatants of GBM cells (D) treated with CTRL or GW4869, and (E) transfected with (left) sh-NC or sh-circNEIL3, and (right) ov-NC or ov-circNEIL3. F Different protein bands detected by silver stain for mass spectrometry of the circNEIL3-protein complex pulled down by circNEIL3 junction sense or anti-sense in U251 cells. G qRT-PCR assays showing the relative expression of immunosuppressive genes in THP1-differentiated macrophages transfected with ov-NC or ov-circNEIL3. H Confocal microscopy analysis showing the internalization of PKH26-labeled exosomes in GBM cells, scale bar, 50 μm. I Relative expression of circNEIL3 in exosomes collected from GBM cells transfected with si-circNEIL3, blank or ov-circNEIL3. qRT-PCR assays showing the relative expression in THP1-differentiated macrophages (J) treated with exosomes collected from GBM cells transfected with si-circNEIL3, blank or ov-circNEIL3, and (K) transfected with ov-NC or ov-IGF2BP3, and (L) co-transfected with ov-NC or ov-circNEIL3 and si-IGF2BP3 as indicated, *n* = 3. Representative flow cytometry assay showing the proportion of CD11b + CD163+ in THP1 differentiated macrophages (M) transfected with ov-NC or ov-IGF2BP3, and (N) co-transfected with ov-NC or ov-circNEIL3 and si-IGF2BP3 as indicated, n = 3. O Representative IF staining in human glioma tissue microarray showed that the expression of IGF2BP3, YAP1, and CD68 was higher in the circNEIL3 high group than in the low group. Histogram representing statistical proportion data of positive area. All data are presented as the means ± SD, and the ov-circNEIL3 group is indicated as the control in (L) and (N), *P < 0.01, **P < 0.01, ***P < 0.001, ****P < 0.0001.

## Data Availability

All data used in this work can be acquired from the TCGA database (http://cancergenome.nih.gov/), CGGA database (http://www.cgga.org.cn/), and the circRNA sequencing and mRNA sequencing data of our local samples have been deposited in the Genome Sequence Archive (GSA) under accession number CRA002339, and data were released when the paper was published. The processed data are available from the corresponding author upon reasonable request.

## Abbreviations

GBM: Glioblastoma multiforme
TME: Tumor microenvironment
CircRNAs: Circular RNAs
BMDMs: Bone marrow-derived macrophages
ELDA: Extreme limiting dilution assy
RIP: RNA immunoprecipitation
Co-IP: Co-immunoprecipitation
EWSR1: EWS RNA-binding protein 1
IGF2BP3: Insulin-like growth factor 2 mRNA binding protein 3
TCGA: The Cancer Genome Atlas
CGGA: Chinese Glioma Genome Atlas
IHC: Immunohistochemistry
IF: Immunofluorescence
ssGSEA: single-sample GSEA
GSVA: Gene set variation analysis
