# Supplementary material for: EWSR1-induced circNEIL3 promotes glioma progression and exosome-mediated macrophage immunosuppressive polarization via stabilizing IGF2BP3
Source: Mol Cancer. 2022 Jan 14;21:16. doi: 10.1186/s12943-021-01485-6 (PMC8759291; doi:10.1186/s12943-021-01485-6)
Supplement: Supplementary file 2 — Additional file 2: Figure S1. Functional analysis of positive genes correlated with circNEIL3 in our local glioma tissues. A KEGG pathway analysis of the positive regulated genes with circNEIL3 in our local glioma dataset. B GSEA analysis revealed that genes with high circNEIL3 expression group were enriched for pathways of malignant tumors in our local glioma dataset. C GSVA enrichment analysis showing the activation status of biological pathways in the circNEIL3-high and circNEIL3-low groups. The heatmap was used to visualize these biological processes. Yellow represents activated pathways, black represents moderately activated pathways, and blue represents inhibited pathways. Figure S2. circNEIL3 promotes gliomagenesis in vitro. qRT-PCR assays showing the expression of (left) circNEIL3 and mRNA NEIl3(right) in GBM cells and GSCs transfected with (A) si-NC or si-circNEIL3, and (B) ov-NC or ov-circNEIL3, n = 3. Representative EDU assay images showing the proliferation ability of GBM cells transfected with (C) si-NC or si-circNEIL3, and (D) ov-NC or ov-circNEIL3. Quantification histogram represented relative cell numbers, n = 3, scale bar, 200um. Representative colony formation assays showing the proliferation ability of GBM cells transfected with (E) si-NC or si-circNEIL3, and (F) ov-NC or ov-circNEIL3. Quantification histogram represented relative cell numbers, n = 3. G Representative images of tumor spheres formation assays showing the tumor spheres formation ability of GSCs transfected with (G) si-NC or si-circNEIL3, and (H) ov-NC or ov-circNEIL3. Quantification histogram represented average sphere dimeter, n = 3, scale bar, 200um. limiting dilution assay showing the self-renewal ability of GSCs transfected with (I) si-NC or si-circNEIL3, and (J) ov-NC or ov-circNEIL3. K Represented H&E staining images for a subgroup of animal sacrificed simultaneously in each group, n = 5 for each group, scale bar, 10 μm. All data are presented as the means ± SD, *P < 0.01, **P [file 12943_2021_1485_MOESM2_ESM.docx]

**Supplementary Figures**


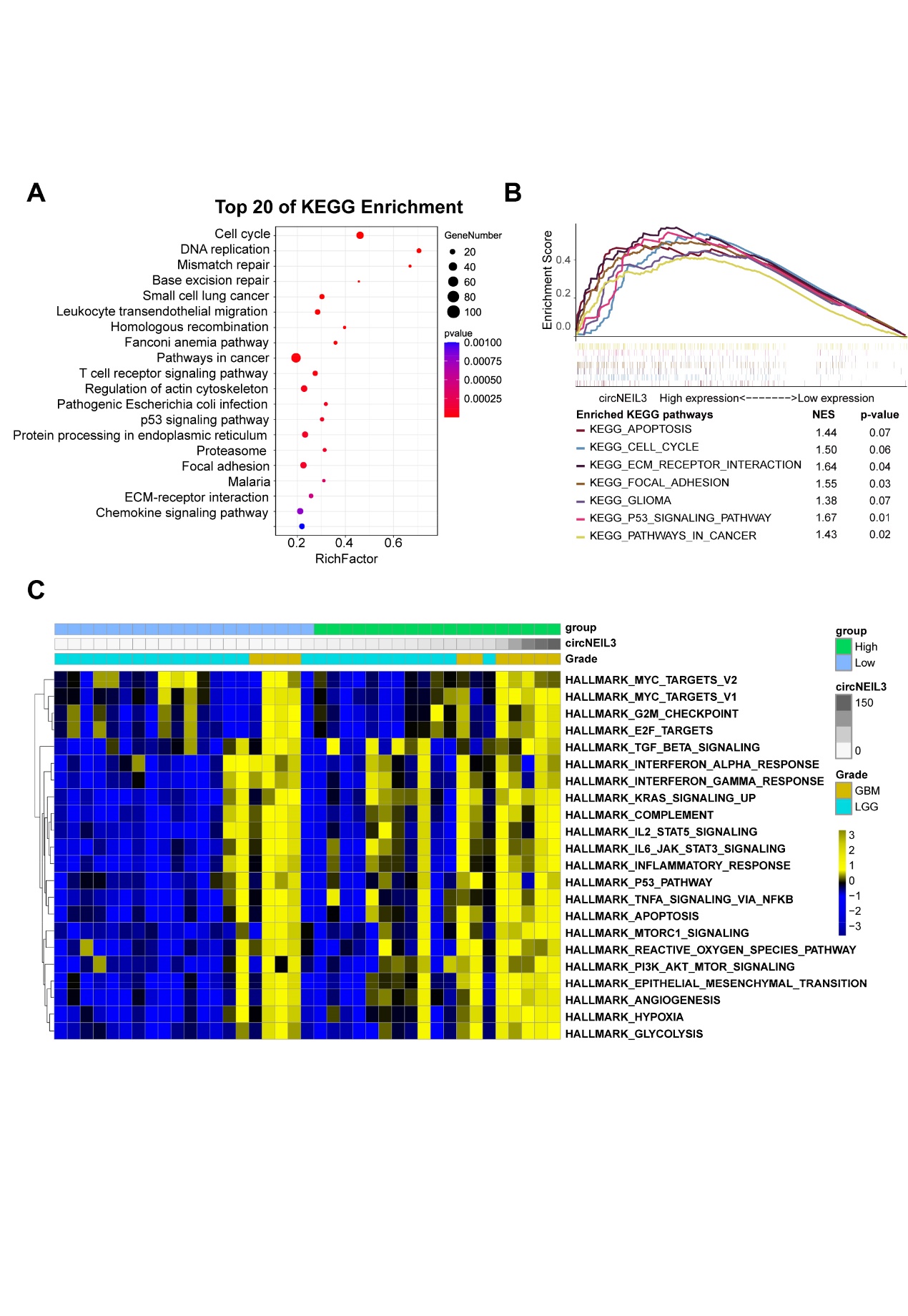


**Fig.S1 Functional analysis of positive genes correlated with circNEIL3 in ourlocal glioma tissues. A** KEGG pathway analysis of the positive regulated genes with circNEIL3 in our local glioma dataset. **B** GSEA analysis revealed that genes with high circNEIL3 expression group were enriched for pathways of malignant tumors in our local glioma dataset. **C** GSVA enrichment analysis showing the activation status of biological pathways in the circNEIL3-high and circNEIL3-low groups. The heatmap was used to visualize these biological processes. Yellow represents activated pathways, black represents moderately activated pathways, and blue represents inhibited pathways.


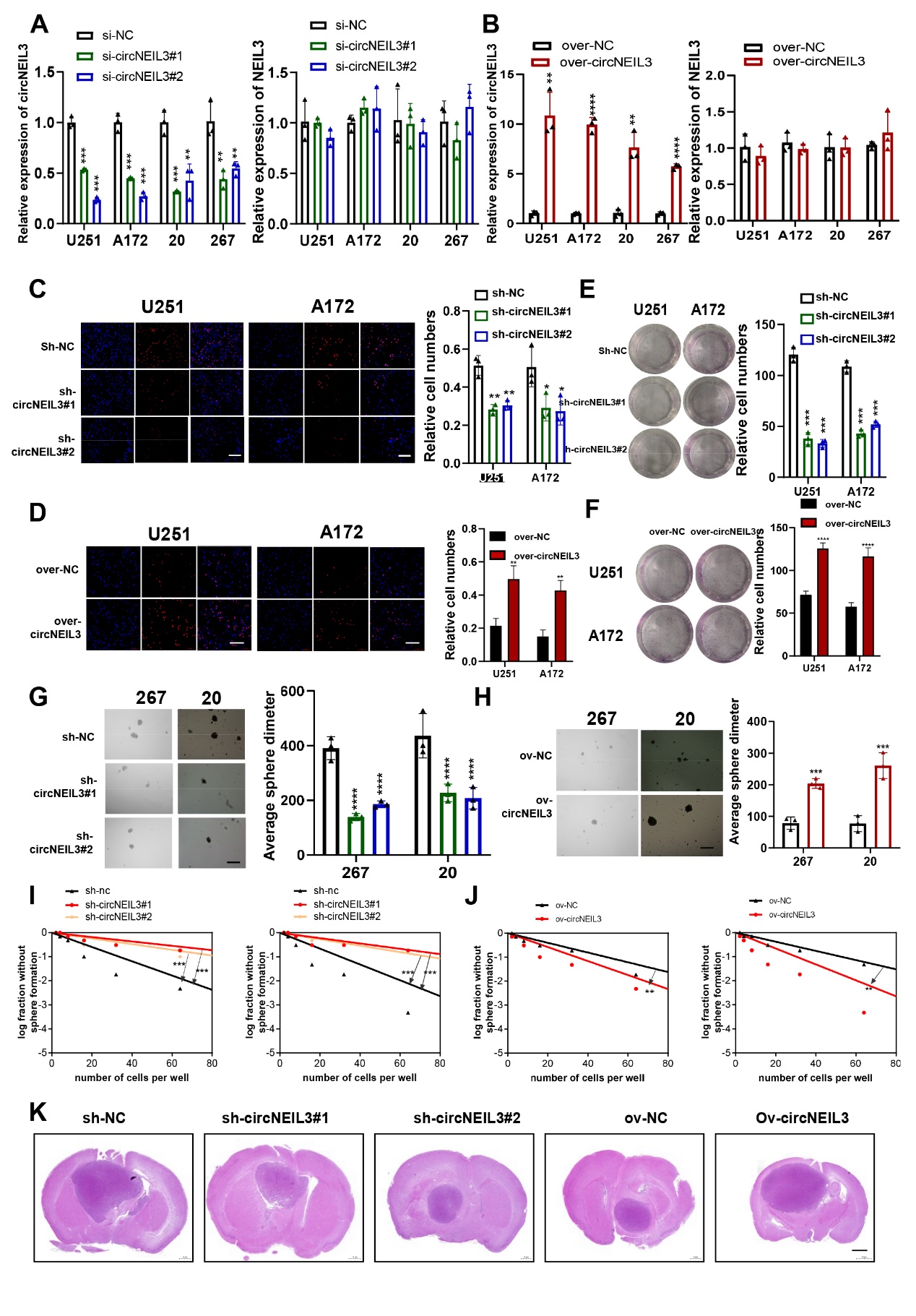


**Fig.S2 circNEIL3 promotes gliomagenesis in vitro.** qRT-PCR assays showing the expression of (left) circNEIL3 and mRNA NEIl3(right) in GBM cells and GSCs transfected with **(A)** si-NC or si-circNEIL3, and **(B)** ov-NC or ov-circNEIL3**, n=3.** Representative EDU assay images showing the proliferation ability of GBM cells transfected with **(C)** si-NC or si-circNEIL3, and **(D)** ov-NC or ov-circNEIL3. Quantification histogram represented relative cell numbers, n=3, scale bar, 200um. Representative colony formation assays showing the proliferation ability of GBM cells transfected with **(E)** si-NC or si-circNEIL3, and **(F)** ov-NC or ov-circNEIL3. Quantification histogram represented relative cell numbers, n=3. G Representative images of tumor spheres formation assays showing the tumor spheres formation ability of GSCs transfected with **(G)** si-NC or si-circNEIL3, and **(H)** ov-NC or ov-circNEIL3. Quantification histogram represented average sphere dimeter, n=3, scale bar, 200um. limiting dilution assay showing the self-renewal ability of GSCs transfected with **(I)** si-NC or si-circNEIL3, and **(J)** ov-NC or ov-circNEIL3. **K** Represented H&E staining images for a subgroup of animal sacrificed simultaneously in each group, n=5 for each group, scale bar, 10μm. All data are presented as the means ± SD, *P< 0.01, **P< 0.01, ***P<0.001, ****P<0.0001.


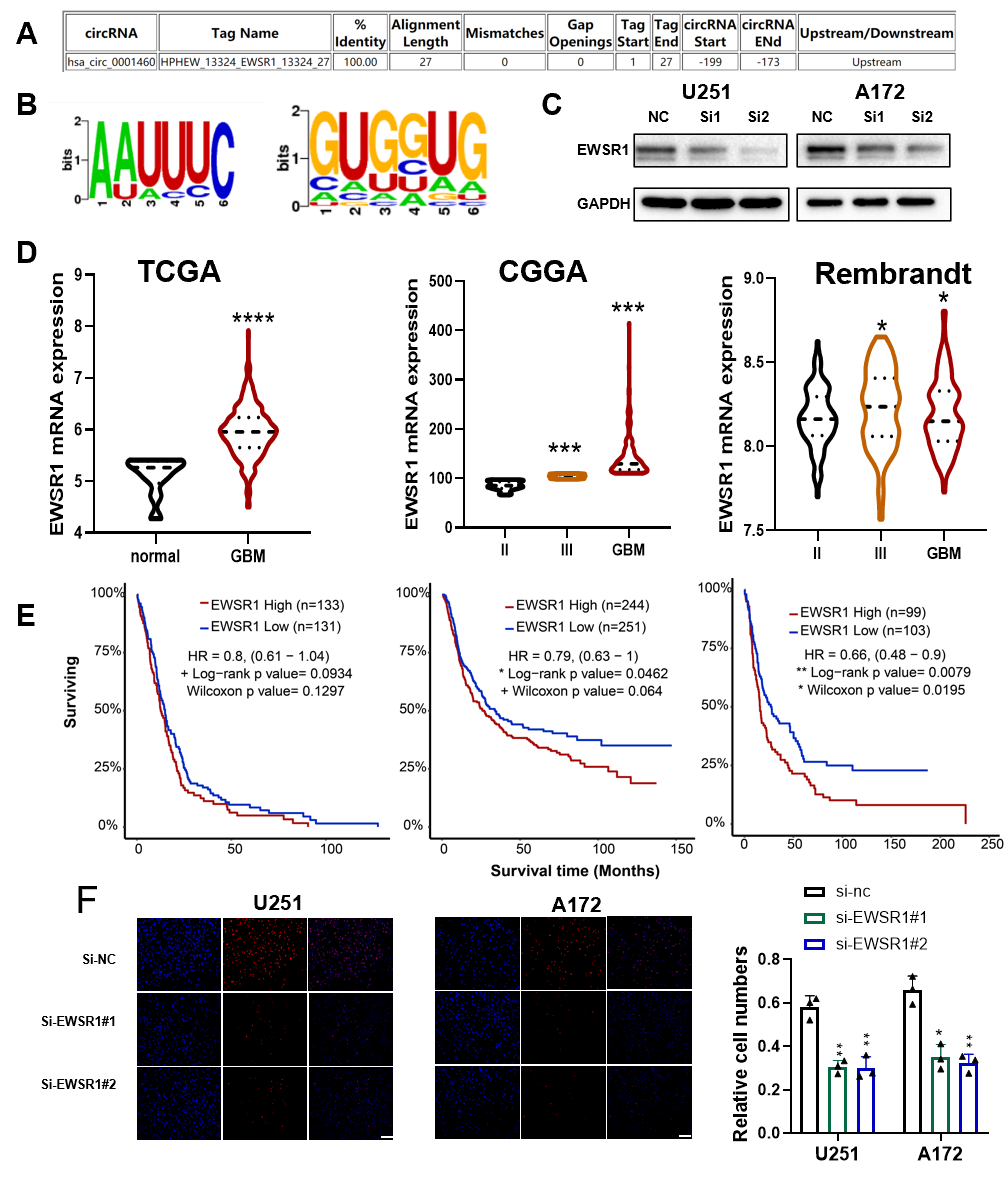


**Fig.S3 EWSR1 promotes the biogenesis of circNEIL3. A** The binding sites of EWSR1 were predicted in the upstream and downstream region of NEIL3 pre-mRNA using circinteratome database. **B** The binding motif of EWSR1 were predicted in the upstream and downstream region of NEIL3 pre-mRNA using starbase database. **C** Western blot assay showing the expression of EWSR1 in GBM cells transfected with si-NC or siEWSR1. **D** The relative expression of EWSR1 in TCGA, CGGA and Rembrandt datasets. **E** Kaplan–Meier survival curves for EWSR1 in TCGA, CGGA and Rembrandt datasets. **F** Representative EDU assays showing the proliferation ability of GBM cells transfected with si-NC or si-EWSR1. Quantification histogram represented relative cell numbers, n=3, scale bar, 200um. All data are presented as the means ± SD, *P< 0.01, **P< 0.01, ***P<0.001, ****P<0.0001.


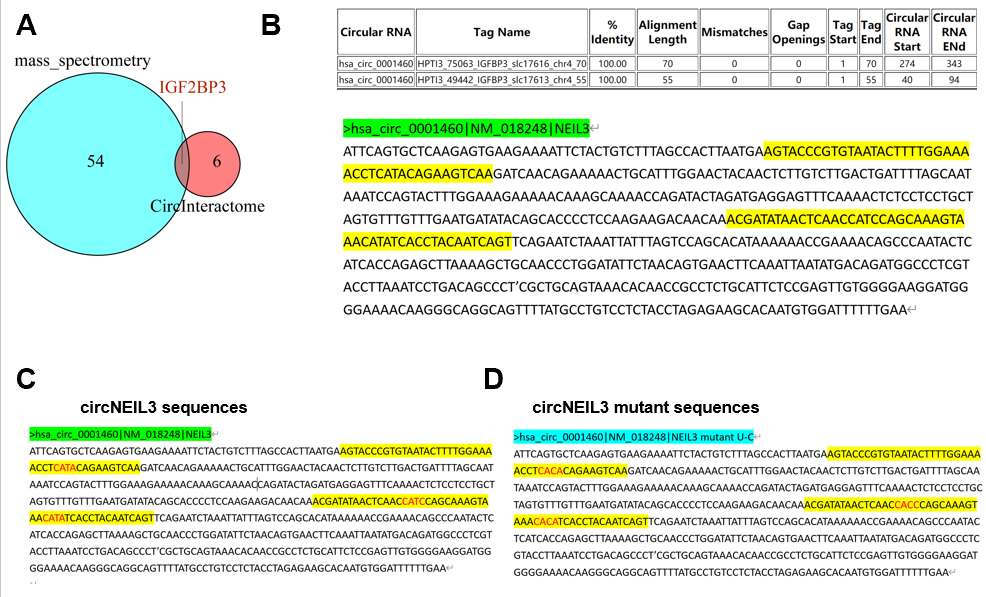


**Fig. S4 CircNEIL3 physically interacts with IGF2BP3. A** Venn diagram showing the overlapping of interacted proteins with circNEIL3 between our mass spectrometric data and prot.eins predicted in CircInteractome database. **B** The sequences that may bind to IGF2BP3 protein predicted in CircInteractome database. **C and D** Sequences of circNEIL3 and circNEIL3 mutant as indicated.


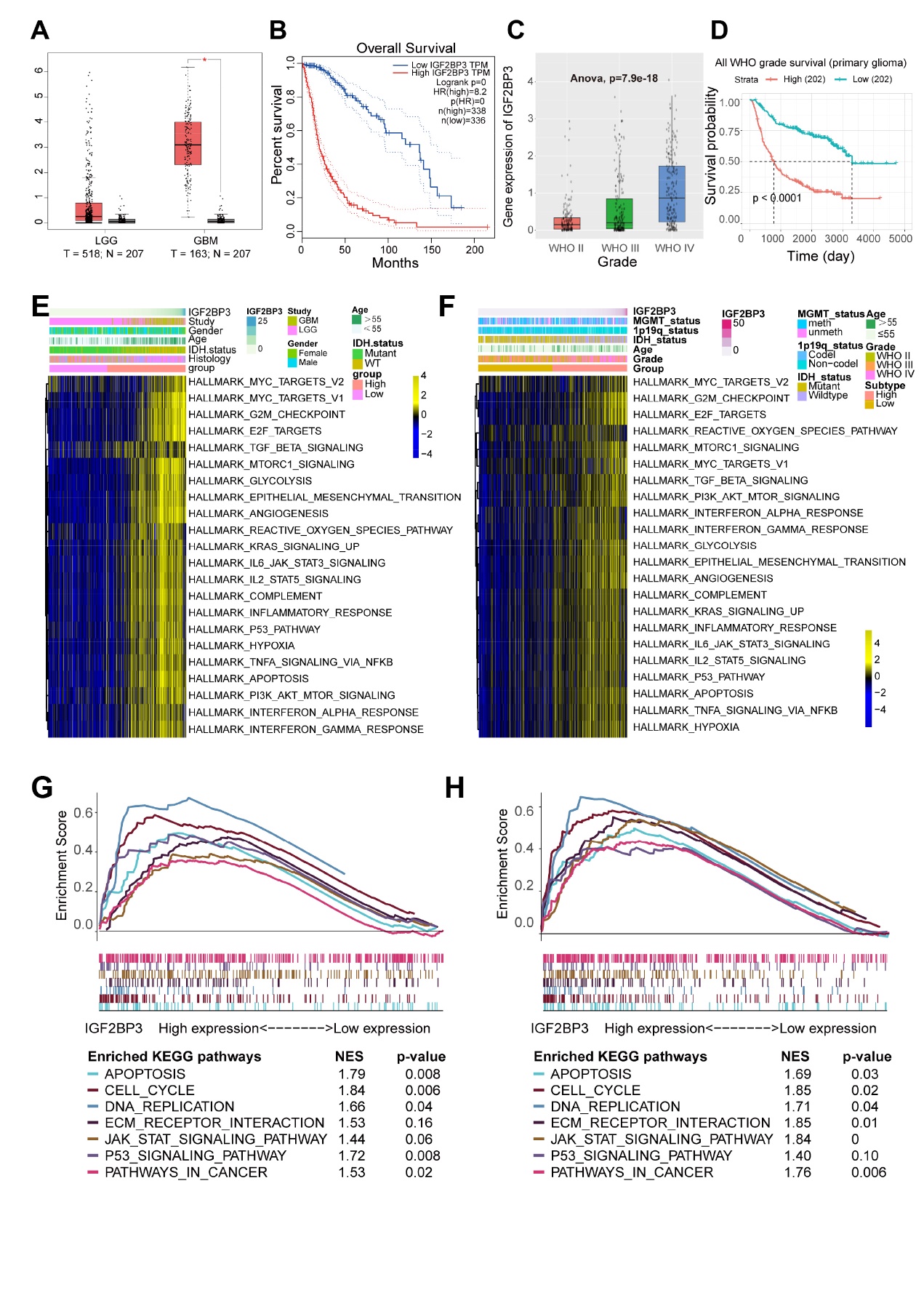


**Fig. S5 IGF2BP3 is correlated with carcinogenic activation pathways in TCGA and CGGA datasets. A** The GEPIA database showing the relative expression of IGF2BP3 in TCGA glioma tissues compared with GETx normal brain. **B** Kaplan–Meier survival curves for IGF2BP3 in TCGA glioma dataset. **C** Relative expression of IGF2BP3 in CGGA glioma dataset. **B** Kaplan–Meier survival curves for IGF2BP3 in CGGA glioma dataset. GSVA enrichment analysis showing the activation status of biological pathways in the IGF2BP3-high and IGF2BP3-low groups in **(E)** TCGA glioma dataset, and **(F)** CGGA glioma dataset. A heatmap was used to visualize these biological processes. Yellow represents activated pathways, black represents moderately activated pathways, and blue represents inhibited pathways. GSEA analysis revealed that genes with high IGF2BP3 expression group were enriched for pathways of malignant tumors in **(G)** TCGA glioma dataset, and **(H)** CGGA glioma dataset.


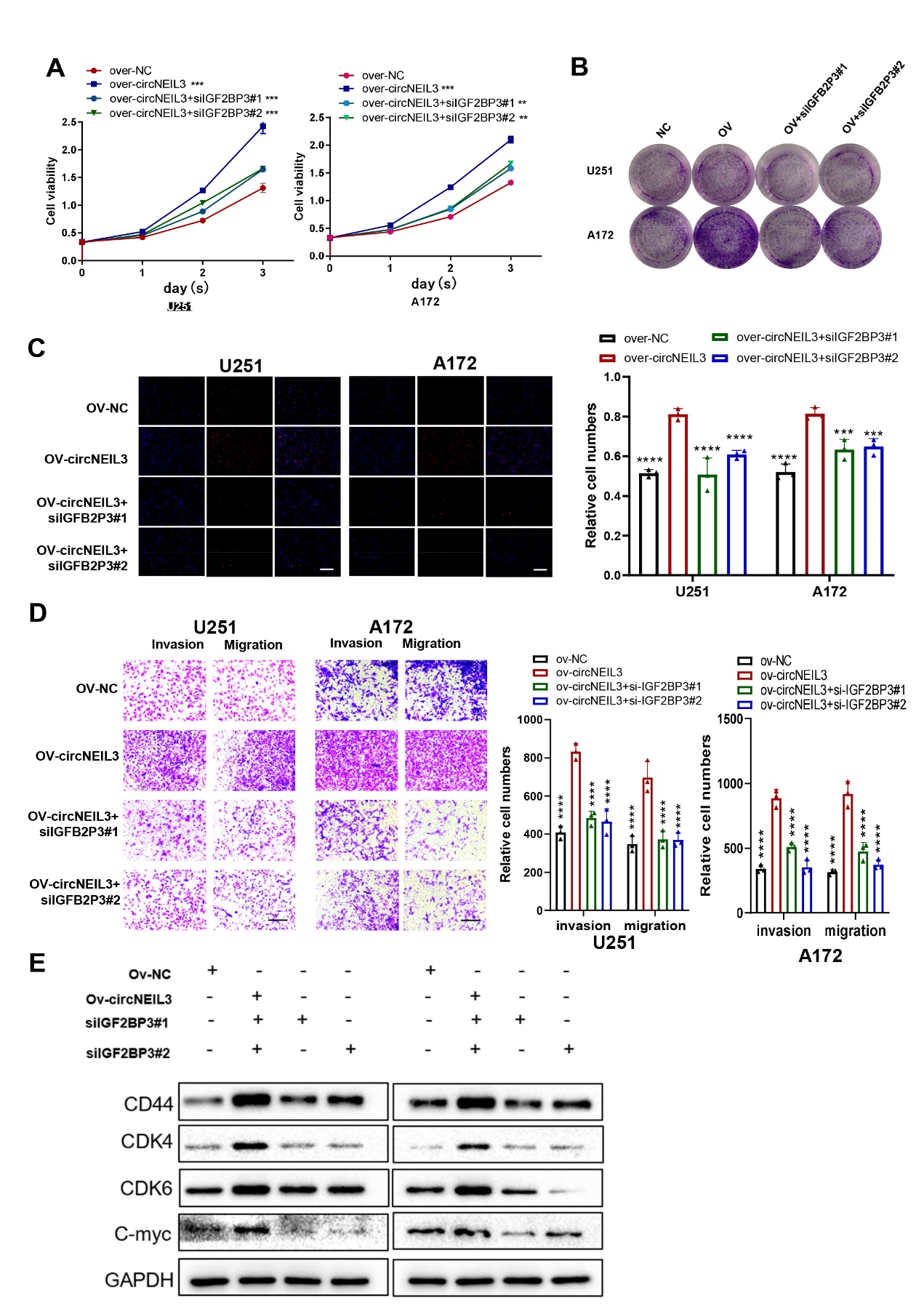


**Fig. S6 circNEIL3 regulated the malignant progression of glioma via destabilizing IGF2BP3 proteins. (A)** CCK8 assays, and **(B)** Representative colony formation assays, and **(C)** Representative EDU assays showing the proliferation ability of GBM cells co-transfected with ov-NC or ov-circNEIL3 and si-IGF2BP3 as indicated. Quantification histogram represented relative cell numbers, n=3, scale bar, 200μm. **D** Representative transwell migration and invasion assays showing the migration and invasion ability of GBM cells co-transfected with ov-NC or ov-circNEIL3 and si-IGF2BP3 as indicated. Quantification histogram represented relative cell numbers, n=3, scale bar, 200um. **E** Western blot assays showing the expression of CD44, CDK4, CDK6 and c-MYC in GBM cells co-transfected with ov-NC or ov-circNEIL3 and si-IGF2BP3 as indicated. All data are presented as the means ± SD, and the ov-circNEIL3 group is indicated as the control, **P< 0.01, ***P<0.001, ****P<0.0001.


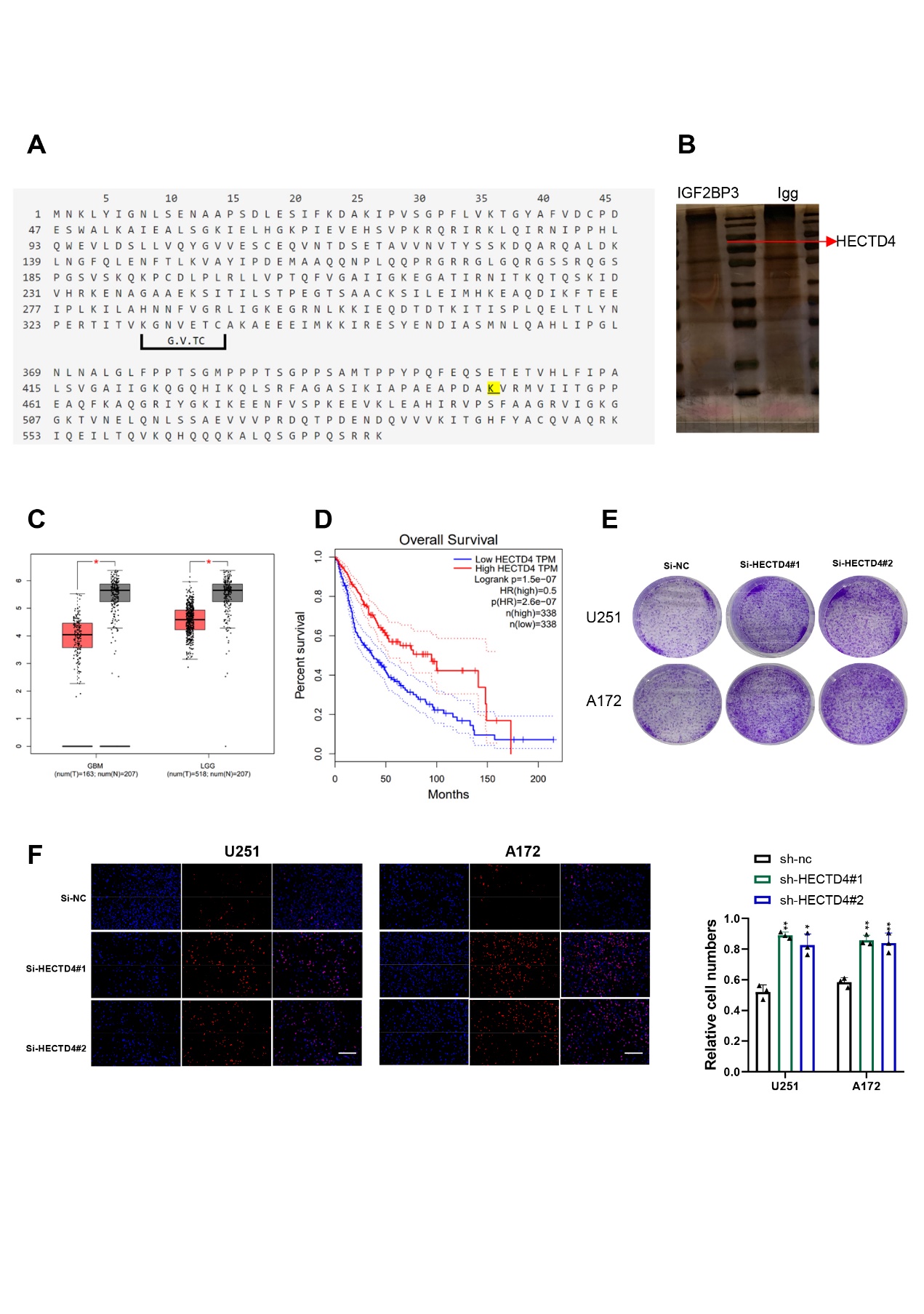


**Fig.S7 circNEIL3 stabilizes IGF2BP3 protein by preventing HECTD4-mediated ubiquitination. A** The ubiquitination site of IGF2BP3 predicted via Ubibrowsers database. **B** Co-IP assays were performed to identify the IGF2BP3-protein complex by IGF2BP3 antibody in GBM cells. **C** The GEPIA database showing the relative expression of HECTD4 in glioma tissues compared with GETx normal brain tissues. **D** Kaplan–Meier survival curves for IGF2BP3 in TCGA glioma dataset. **E** Colony formation assays showing the proliferation ability of GBM cells transfected with si-NC or si-HECTD4. **F** Representative EDU assays showing the proliferation ability of GBM cells transfected with si-NC or si-HECTD4. Quantification histogram represented relative cell numbers, n=3, scale bar, 200um. All data are presented as the means ± SD, and the ov-circNEIL3 group is indicated as the control, *P< 0.05, **P< 0.01.


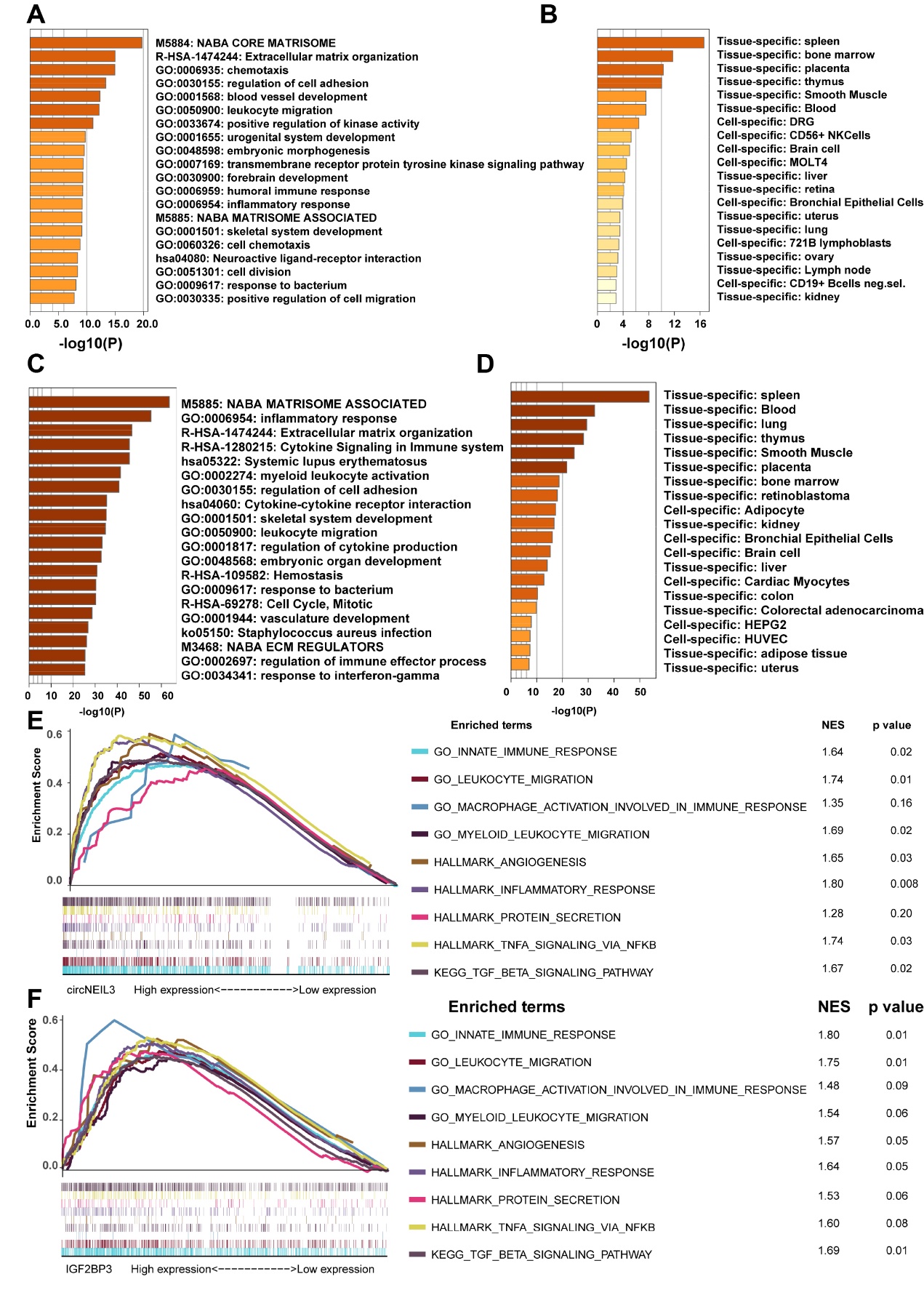


**Fig.S8 The genes that upregulated in circNEIL3 and IGF2BP3 high expression group were enriched in immune response- and stromal-related pathways.** Bar graph of **(A)** enriched terms, colored by p-values, and **(B)** Summary of enrichment analysis in PaGenBase across upregulated genes in circNEIL3 high group in our local glioma samples. Bar graph of **(C)** enriched terms, colored by p-values, and **(D)** Summary of enrichment analysis in PaGenBase database across upregulated genes in IGF2BP3 high group in TCGA glioma samples. GSEA analysis revealed that genes with **(E)** high circNEIL3 expression group in our local glioma samples, and **(F)** high IGF2BP3 expression in TCGA glioma samples were enriched for immune response related pathways.


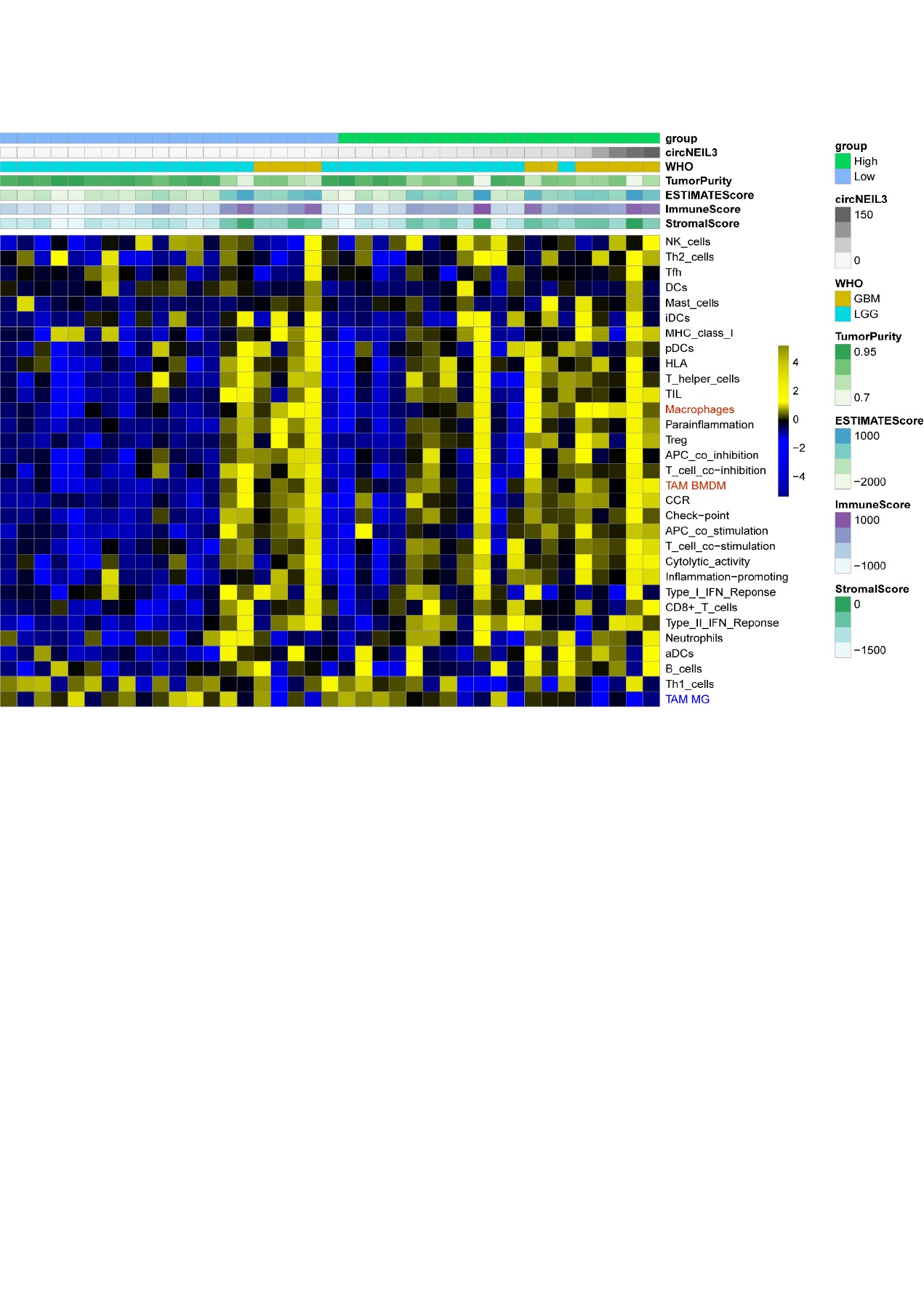


**Fig. S9 CircNEIL3 is correlated with immunophenotypes and TME landscapes.** GSVA enrichment analysis showing the enrichment scores of immune cell types and immune-related function related gene sets in the circNEIL3-high and circNEIL3-low groups. A heatmap was used to visualize these immune characteristics between the circNEIL3 high and low expression groups; yellow represents a high enrichment level, black represents a median enrichment level, and blue represents a low enrichment level.


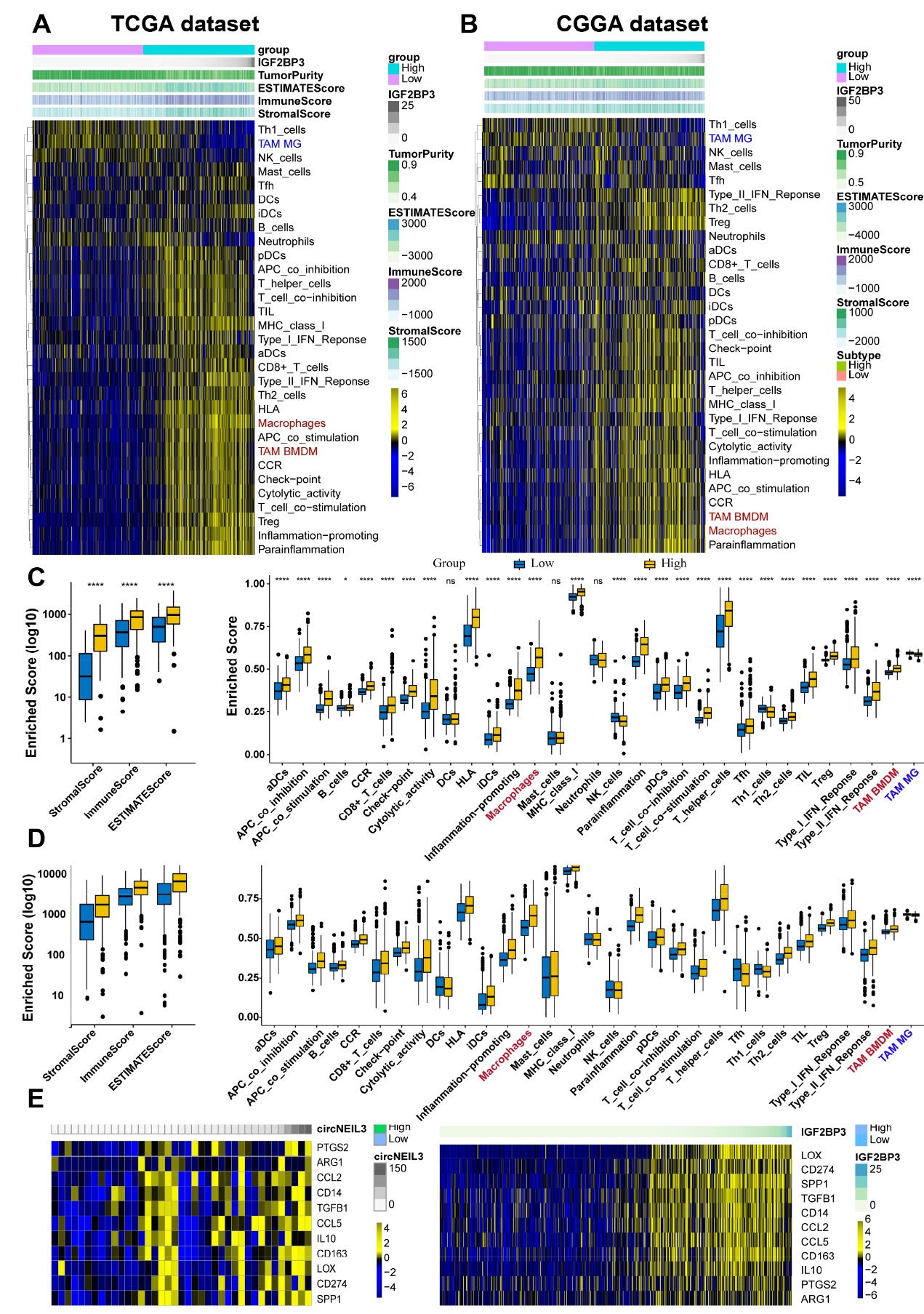


**Fig. S10 IGF2BP3 is correlated with immunophenotypes and TME landscapes.** GSVA enrichment analysis showing the enrichment scores of immune cell types and immune-related function related gene sets in the IGF2BP3-high and IGF2BP3-low groups in **(A)** TCGA glioma dataset, and **(B)** CGGA glioma dataset. A heatmap was used to visualize these immune characteristics between the IGF2BP3 high and low expression groups; yellow represents a high enrichment level, black represents a median enrichment level, and blue represents a low enrichment level. The abundance of immune/stromal score, each TME infiltrating cells and regulators in IGF2BP3 high and low groups in **(C)** TCGA dataset, and **(D)** CGGA dataset. **E** GSVA enrichment analysis showing the immunosuppressive genes in the (left) circNEIL3-high and circNEIL3-low groups in our local glioma samples, and (right) IGF2BP3-high and IGF2BP3-low groups in TCGA glioma samples. The statistical p-value was calculated using the nonparametric Wilcoxon test, ns, P>0.05, *P<0.05, **P< 0.01, ***P<0.001, ****P<0.0001.


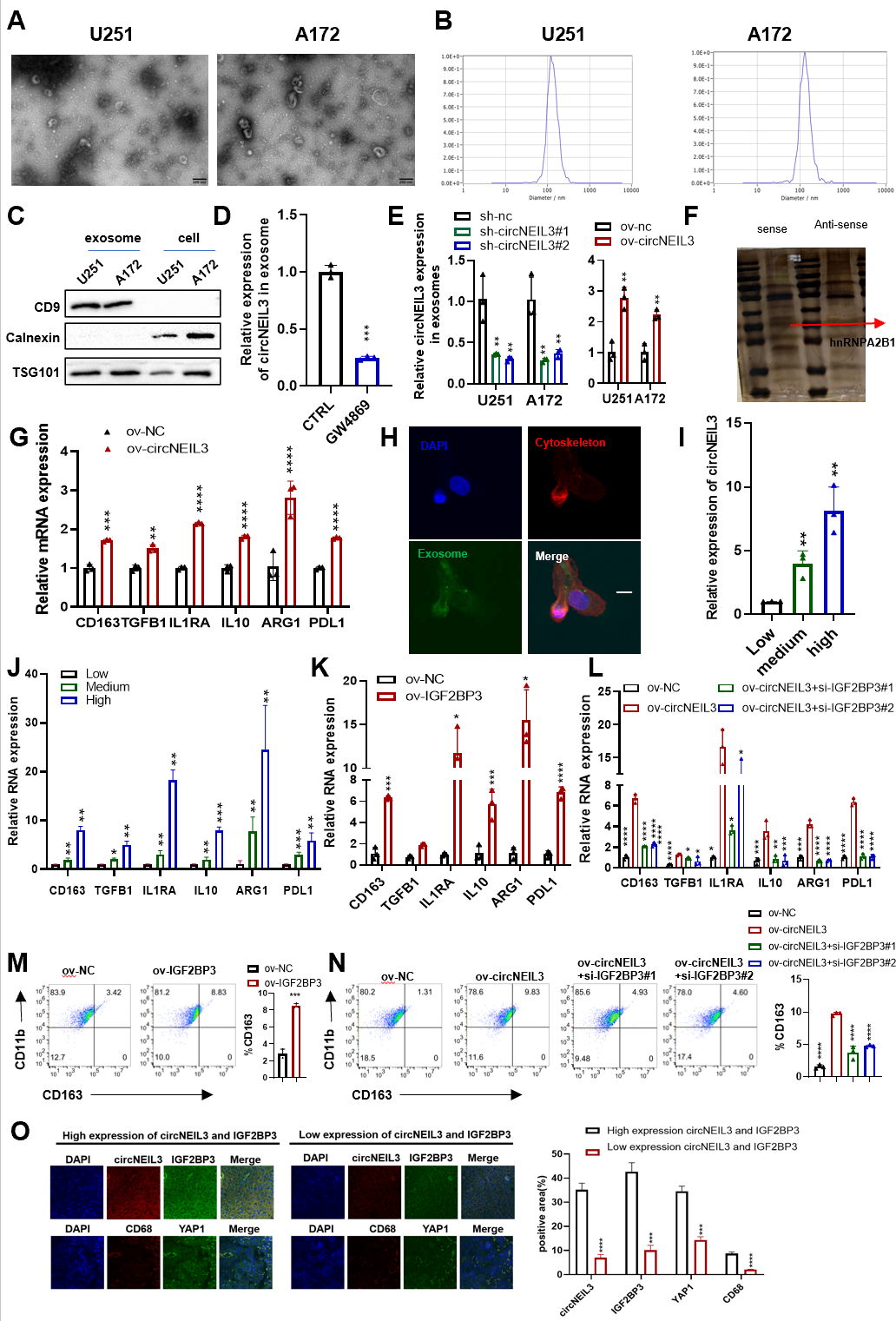


**Fig.S11 Exosomes deliver circNEIL3 to TAMs, thereby enabling them to acquire immunosuppressive properties via stabilizing IGF2BP3. A** Transmission electron microscopy detected the exosomes isolated from supernatants of GBM cells via ultracentrifugation, scale bar, 200nm. **B** NanoSight particletracking analysis of the size distributions and number of exosomes. **C** Western blot assay showing the exosomes markers (CD9, Calnexin and TSG101) in exosomes isolated from supernatants of GBM cells via ultracentrifugation and corresponding GBM cells. qRT-PCR assay showing the expression of circNEIL3 in exosomes isolated from supernatants of GBM cells **(D)** treated with CTRL or GW4869, and **(E)** transfected with (left) sh-NC or sh-circNEIL3, and (right) ov-NC or ov-circNEIL3. **F** Different protein bands detected by silver stain for mass spectrometry of the circNEIL3-protein complex pulled down by circNEIL3 junction sense or anti-sense in U251 cells. **G** qRT-PCR assays showing the relative expression of immunosuppressive genes in THP1-differentiated macrophages transfected with ov-NC or ov-circNEIL3. **H** Confocal microscopy analysis showing the internalization of PKH26-labeled exosomes in GBM cells, scale bar, 50 um. **I** Relative expression of circNEIL3 in exosomes collected from GBM cells transfected with si-circNEIL3, blank or ov-circNEIL3. qRT-PCR assays showing the relative expression in THP1-differentiated macrophages **(J)** treated with exosomes collected from GBM cells transfected with si-circNEIL3, blank or ov-circNEIL3, and **(K)** transfected with ov-NC or ov-IGF2BP3, and **(L)** co-transfected with ov-NC or ov-circNEIL3 and si-IGF2BP3 as indicated, n=3. Representative flow cytometry assay showing the proportion of CD11b+ CD163+ in THP1 differentiated macrophages **(M)** transfected with ov-NC or ov-IGF2BP3, and **(N)** co-transfected with ov-NC or ov-circNEIL3 and si-IGF2BP3 as indicated, n=3. **O** RepresentativeIF staining in human glioma tissue microarray showed that the expression of IGF2BP3, YAP1, and CD68 was higher in the circNEIL3 high group than in the low group. Histogram representing statistical proportion data of positive area. All data are presented as the means ± SD, and the ov-circNEIL3 group is indicated as the control in (L) and (N), *P< 0.01, **P< 0.01, ***P<0.001, ****P<0.0001.
